# 2-D Molecular Alloy Ru–M (M = Cu, Ag, and Au) Carbonyl Clusters: Synthesis, Molecular Structure, Catalysis, and Computational Studies

**DOI:** 10.1021/acs.inorgchem.2c02099

**Published:** 2022-09-07

**Authors:** Cristiana Cesari, Marco Bortoluzzi, Francesca Forti, Lisa Gubbels, Cristina Femoni, Maria Carmela Iapalucci, Stefano Zacchini

**Affiliations:** † Dipartimento di Chimica Industriale “Toso Montanari”, Università di Bologna, Viale Risorgimento 4, 40136 Bologna, Italy; ‡ Center for Chemical Catalysis—C3, University of Bologna, Viale Risorgimento 4, 40136 Bologna, Italy; § Dipartimento di Scienze Molecolari e Nanosistemi, Ca’ Foscari University of Venice, Via Torino 155, 30175 Mestre (Ve), Italy

## Abstract

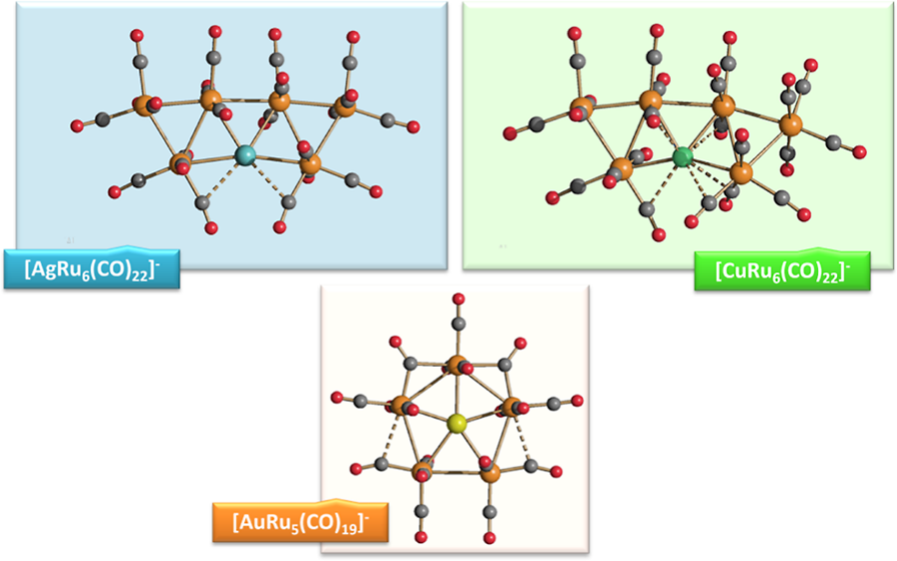

The reactions of [HRu_3_(CO)_11_]^−^ (**1**) with M(I) (M = Cu, Ag, and Au) compounds such as [Cu(CH_3_CN)_4_][BF_4_], AgNO_3_, and Au(Et_2_S)Cl afford the 2-D molecular alloy clusters [CuRu_6_(CO)_22_]^−^ (**2**), [AgRu_6_(CO)_22_]^−^ (**3**), and [AuRu_5_(CO)_19_]^−^ (**4**), respectively. The reactions of **2–4** with PPh_3_ result in mixtures of products, among which [Cu_2_Ru_8_(CO)_26_]^2–^ (**5**), Ru_4_(CO)_12_(CuPPh_3_)_4_ (**6**), Ru_4_(CO)_12_(AgPPh_3_)_4_ (**7**), Ru(CO)_3_(PPh_3_)_2_ (**8**), and HRu_3_(OH)(CO)_7_(PPh_3_)_3_ (**9**) have been isolated and characterized. The molecular structures of **2–6** and **9** have been determined by single-crystal X-ray diffraction. The metal–metal bonding within **2–5** has been computationally investigated by density functional theory methods. In addition, the [NEt_4_]^+^ salts of **2–4** have been tested as catalyst precursors for transfer hydrogenation on the model substrate 4-fluoroacetophenone using ^*i*^PrOH as a solvent and a hydrogen source.

## Abstract 2

Heterometallic Ru−M (M = Cu, Ag, and Au) carbonyl clusters possessing a 2-D metal core have been investigated by structural and computational methods and tested as catalyst precursors for transfer hydrogenation.

## 1 Introduction

Molecular clusters usually adopt 3-D structures that consist of tridimensional metal cores, such as tetrahedron, octahedron, icosahedron, and larger polyhedral, as well as more complex and irregular structures.^1,2^ Further growth in a 3-D mode results in molecular nanoclusters and larger metal nanoparticles.^3−10^ Alternatively, molecular clusters may adopt 2-D structures that consist of a planar or almost planar arrangement of metal atoms.^11,12^ Even if 2-D clusters are rarer than 3-D ones, representative examples are found within heterometallic (alloy) molecular carbonyl clusters such as [M_3_Fe_3_(CO)_12_]^3–^ (M = Cu, Ag, and Au),^13,14^ [M_4_Fe_4_(CO)_16_]^4–^ (M = Ag and Au),^15,16^ [M_5_Fe_4_(CO)_16_]^3–^ (M = Cu, Ag, and Au),^15−17^ [Os_9_Hg_3_(CO)_30_],^18^ and [IrRu_6_(CO)_23_]^−^.^19^ It should be mentioned that a few cases of the 1-D growth path of carbonyl clusters have been reported,^20^ being homoleptic and heteroleptic Chini clusters the most astonishing examples.^21−23^ Compared to 3-D clusters, the nuclearity of 2-D clusters reported so far is rather limited. Nonetheless, they are attractive from a structural point of view as models of metal surfaces and monolayers.^24−26^

Heterometallic complexes and clusters containing polar metal–metal interactions are attracting interest for the activation of small molecules and catalytic applications.^27−31^ Examples include C–H functionalization, carbonylation, hydrogenation,^32^ as well as ammonia-borane dehydrogenation.^33^ Like ammonia-borane, alcohols such as iso-propanol also contain at the same time both hydridic-like (C–H) and protic (O–H) hydrogens, suitable for activation via interaction with a polar metal–metal bond. Thus, heterometallic clusters such as Ru–M (M = Cu, Ag, and Au) might be active as catalysts for transfer hydrogenation reactions.^34^

Within this framework, herein, we report the synthesis of the 2-D molecular alloy carbonyl clusters [CuRu_6_(CO)_22_]^−^, [AgRu_6_(CO)_22_]^−^, and [AuRu_5_(CO)_19_]^−^. Their molecular structures have been determined by single-crystal X-ray diffraction (SC-XRD), and the metal–metal bonding has been analyzed by computational methods. Moreover, some preliminary results on their use as pre-catalysts in transfer hydrogenation reactions are reported.

## 2 Results and Discussion

### 2.1 Synthesis, Molecular Structure, and Computational Analysis of [MRu_6_(CO)_22_]^−^ (M = Cu and Ag)

**Scheme 1:**
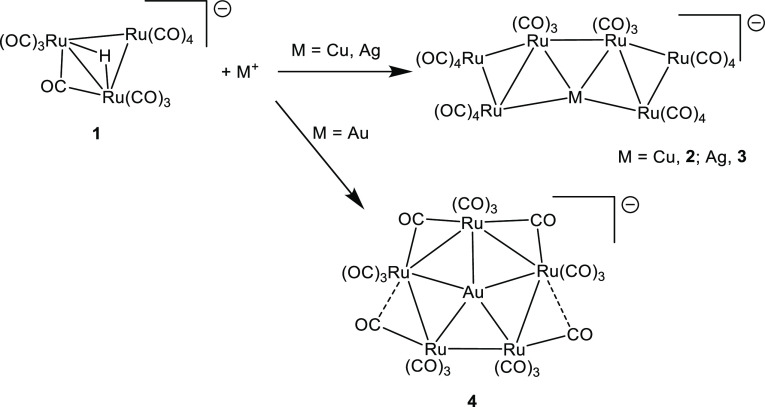
Synthesis of [CuRu_6_(CO)_22_]^−^ (**2**), [AgRu_6_(CO)_22_]^−^ (**3**), and [AuRu_5_(CO)_19_]^−^ (**4**) from [HRu_3_(CO)_11_]^−^ (**1**)

The reaction of [NEt_4_][HRu_3_(CO)_11_] ([NEt_4_][**1**]) in CH_2_Cl_2_ with 0.5 mole equiv of [Cu(CH_3_CN)_4_][BF_4_] or AgNO_3_ results in the formation of [NEt_4_][CuRu_6_(CO)_22_] ([NEt_4_][**2**]) and [NEt_4_][AgRu_6_(CO)_22_] ([NEt_4_][**3**]), respectively (Scheme 1). In contrast, [NEt_4_][AuRu_5_(CO)_19_] ([NEt_4_][**4**]) is obtained when employing [NEt_4_][**1**] and Au(Et_2_S)Cl under similar experimental conditions. In all cases, the M(I) reagent must be added slowly, thus avoiding any excess, in order to limit the formation of Ru_3_(CO)_12_ as the side product. New compounds [NEt_4_][**2**], [NEt_4_][**3**], and [NEt_4_][**4**] have been characterized by infrared (IR) spectroscopy (Figures S1–S6 in the Supporting Information) and SC-XRD.

**Figure 1:**
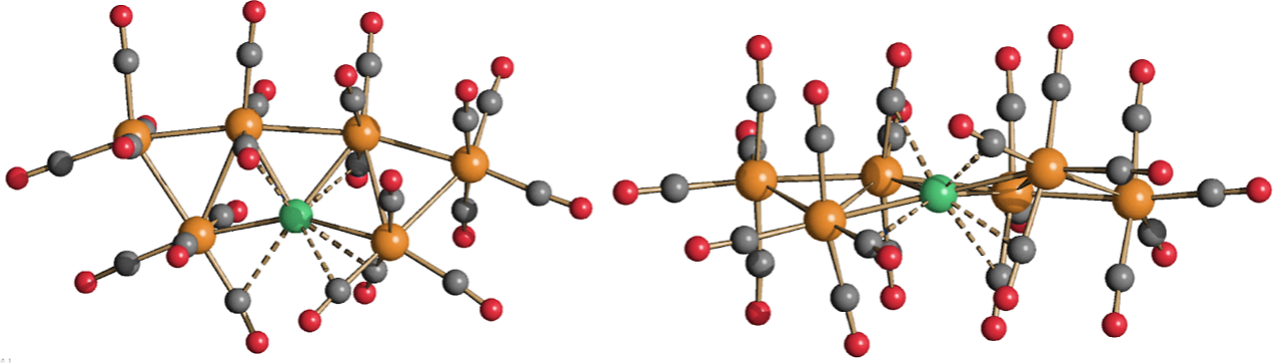
Molecular structure of [CuRu_6_(CO)_22_]^−^ (**2**) (orange Ru; green Cu; red O; gray C). Cu···C(O) contacts [2.49–2.87 Å] are represented as fragmented lines.

**Figure 2:**
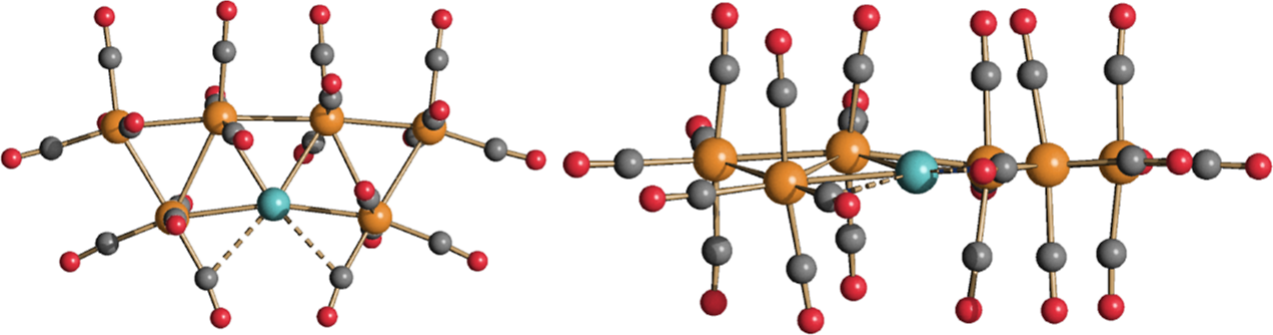
Molecular structure of [AgRu_6_(CO)_22_]^−^ (**3**) (orange Ru; cyan Ag; red O; gray C). Ag···C(O) contacts [2.74 Å] are represented as fragmented lines.

The solid-state structures of [NEt_4_][**2**] and [NEt_4_][**3**] consist of ionic packings of [NEt_4_]^+^ cations and [MRu_6_(CO)_22_]^−^ (M = Cu and Ag) anions. The cluster anions are composed of two Ru_3_ triangular units joined by a direct Ru–Ru interaction (Figures 1 and 2, Table 1). The unique M atom bridges these inter-triangular Ru–Ru contacts and, at the same time, one edge of both of the two triangular units. Overall, M forms four M–Ru bonding contacts, resulting in a 2-D MRu_6_ metal core which is completed by 22 terminal CO ligands (11 per each Ru_3_ unit). Some weak M···C(O) contacts are also present, four in **2** [Cu···C(O) 2.49–2.87 Å] and two in **3** [Ag···C(O) 2.74 Å]. The bridge asymmetry parameter (α) of these CO ligands is 0.30–0.49 for **2** and 0.43 for **3** in the range for semi-bridging ligands [α = 0.1–0.6].^35^ Nonetheless, the IR spectra in CH_2_Cl_2_ of **2** [ν_CO_ 2070(m), 2037(sh), 2014(sh), and 1970(m) cm^–1^] and **3** [ν_CO_ 2067(m), 2034(sh), 2022(sh), 2009(vs), and 1970(ms) cm^–1^] display only terminal carbonyls, with no evidence of bridging or semi-bridging ligands. It may be that the M···C(O) contacts are present only in the solid state due to packing effects, or that such interactions are weak and do not affect the IR spectra.^36−38^ Indeed, there is no evidence for bridging (or semi-bridging) carbonyls in the IR spectra recorded in the solid state.

**Table 1:** Main Bond Distances (Å) and Angles (°) of [MRu_6_(CO)_22_]^−^ (M = Cu and Ag)^a^

M	Cu (**2**)	Ag (**3**)
M(1)–Ru(1)	2.596(2)	2.8086(2)
	*(1.08)*	*(1.09)*
M(1)–Ru(6)	2.613(2)	2.8086(2)
	*(1.08)*	*(1.09)*
M(1)–Ru(3)	2.630(2)	2.7876(3)
	*(1.09)*	*(1.08)*
M(1)–Ru(4)	2.580(2)	2.7876(3)
	*(1.07)*	*(1.08)*
Ru(1)–Ru(3)	3.0189(16)	3.0216(3)
	*(1.22)*	*(1.22)*
Ru(4)–Ru(6)	2.9857(16)	3.0216(3)
	*(1.20)*	*(1.22)*
Ru(1)–Ru(2)	2.9136(16)	2.9075(3)
	*(1.17)*	*(1.17)*
Ru(5)–Ru(6)	2.8982(16)	2.9075(3)
	*(1.17)*	*(1.17)*
Ru(2)–Ru(3)	2.8085(15)	2.8257(3)
	*(1.13)*	*(1.14)*
Ru(4)–Ru(5)	2.8041(15)	2.8257(3)
	*(1.13)*	*(1.14)*
Ru(3)–Ru(4)	2.9304(15)	2.9925(4)
	*(1.18)*	*(1.21)*
Ru(1)–M(1)–Ru(6)	155.01(8)	165.417(13)
Ru(3)–M(1)–Ru(4)	68.44(5)	64.927(11)
Ru(1)–M(1)–Ru(3)	70.57(5)	65.359(7)
Ru(4)–M(1)–Ru(6)	70.19(5)	65.359(7)
mean deviation from MRu_6_ least-square plane	0.2897	0.1138

a Cotton’s FSRs are reported in parentheses. Pauling’s atomic radii are employed: Ru 1.241 Å, Cu 1.173 Å, and Ag 1.339 Å. See Scheme 2 for labeling.

The Ag–Ru contacts [2.7876(3)–2.8086(2) Å] of **3** are longer than the Cu–Ru contacts [2.580(2)–2.630(2) Å] of **2** in view of the larger covalent radius of Ag [1.339 Å] as compared to that of Cu [1.173 Å].^39^ Nonetheless, the formal shortness ratios (FSR) of the M–Ru contacts of **3** [FSR 1.08–1.09] and **2** [FSR 1.07–1.09] are very similar.^40^

The larger size of Ag as compared to Cu has two further consequences. First, the inter-triangular Ru–Ru contact of **3** [2.9925(4) Å] is longer than that of **2** [2.9304(15) Å], whereas the intra-triangular Ru–Ru bonding distances of the two clusters are very similar [2.8041(15)–3.0189(16) Å for **2**; 2.8257(3)–3.0216(3) Å for **3**]. Moreover, the mean deviation from the MRu_6_ least-square plane of **2** [0.2897 Å] is more than twice that of **3** [0.1138 Å]. Thus, the metal core of **3** is almost planar, whereas considerable twisting is present in the case of **2** in order to accommodate the smaller Cu^+^ ion.

**Figure 3:**
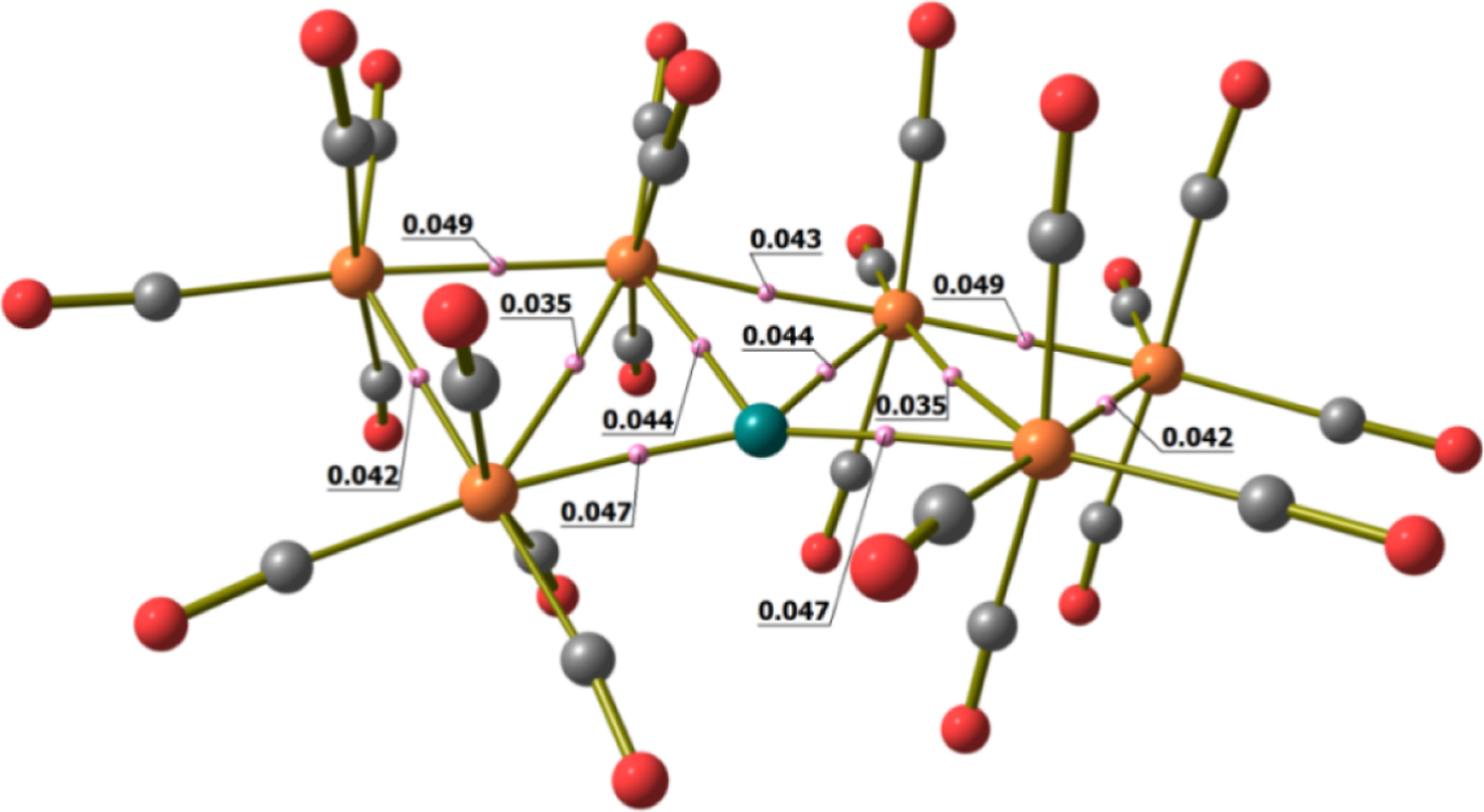
DFT-optimized structure of **2** (orange Ru; green Cu; red O; gray C) with M–M b.c.p.’s and corresponding ρ values (pink, a.u.).

The density functional theory (DFT)-optimized structure of **2** is in good agreement with the experimental data [root-mean-square deviation (RMSD) = 0.359 Å]. The shortest Cu···C distance observed in the X-ray structure is elongated by about 0.48 Å in the computed geometry, and no bond critical point (b.c.p.) was found between the copper center and the terminal carbonyl ligands; therefore, the Cu···C(O) contacts are probably due to packing effects. On the other hand, b.c.p.’s were found for all the Cu–Ru and Ru–Ru bonds, as observable in Figure 3. Selected data are collected in Table 2 and summarized on the basis of the approximate *C*_2_ symmetry of the optimized geometry (*R* = 0.116). In all cases, the negative values of energy density (*E*) and the positive values of the Laplacian of electron density (∇^2^ρ) at b.c.p.’s are in line with Bianchi’s classification of metal–metal bonds.^41^ The different strengths of the Cu–Ru interactions are highlighted in particular by the Wiberg analysis (Table 2), indicating that the Cu(1)–Ru(1)/Cu(1)–Ru(6) bonds are stronger than Cu(1)–Ru(3)/Cu(1)–Ru(4). Both AIM and Wiberg analyses suggest that Ru–Ru bond strengths follow the order Ru(2)–Ru(3)/Ru(4)–Ru(5) > Ru(1)–Ru(2)/Ru(5)–Ru(6) ≈ Ru(3)–Ru(4) > Ru(1)–Ru(3)/Ru(4)–Ru(6) (see Scheme 2 for labeling).

**Scheme 2:**
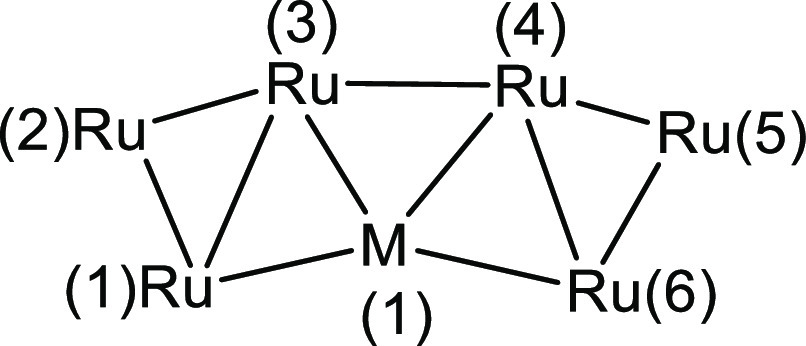
Labeling of [MRu_6_(CO)_22_]^−^ (M = Cu and Ag)

**Table 2:** Selected Average Computed Data (a.u.) at Metal–Metal b.c.p.’s for **2** (ρ = Electron Density; *V* = Potential Energy Density; *E* = Energy Density; ∇^2^ρ = Laplacian of Electron Density) and Wiberg Bond Orders

bond	ρ	*V*	*E*	∇^2^ρ	Wiberg b.o.
Cu(1)–Ru(1)/Cu(1)–Ru(6)	0.047	–0.043	–0.015	0.056	0.347
Cu(1)–Ru(3)/Cu(1)–Ru(4)	0.044	–0.044	–0.013	0.076	0.308
Ru(1)–Ru(2)/Ru(5)–Ru(6)	0.042	–0.030	–0.011	0.027	0.485
Ru(2)–Ru(3)/Ru(4)–Ru(5)	0.049	–0.037	–0.014	0.038	0.583
Ru(1)–Ru(3)/Ru(4)–Ru(6)	0.035	–0.025	–0.008	0.037	0.411
Ru(3)–Ru(4)	0.043	–0.028	–0.011	0.026	0.519

**Figure 4:**
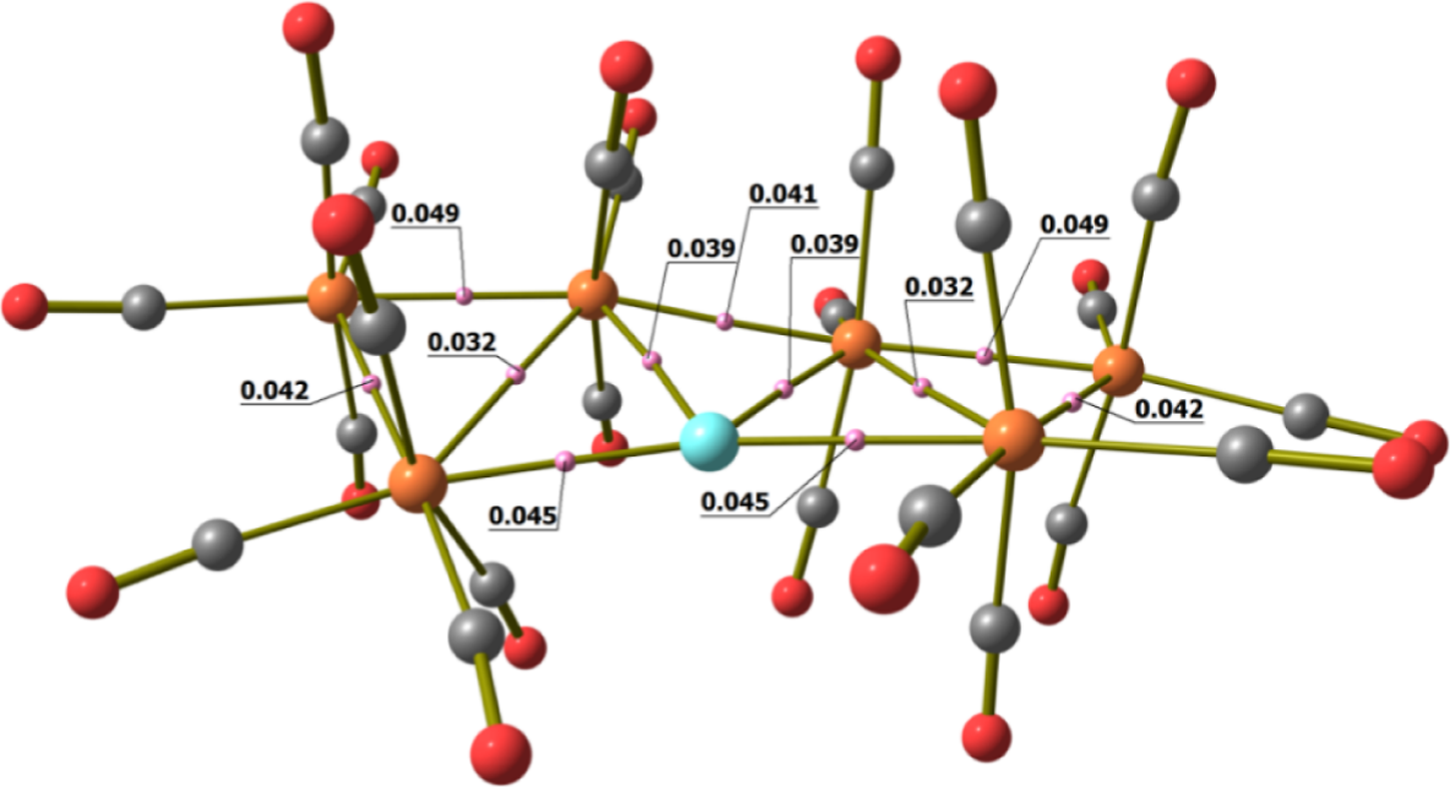
DFT-optimized structure of **3** (orange Ru; cyan Ag; red O; gray C) with M–M b.c.p.’s and corresponding ρ values (pink, a.u.).

As for **2**, the DFT-optimized structure of **3** is in good agreement with the experimental data (RMSD = 0.357 Å). In this case, the optimized geometry has a regular *C*_2_ symmetry (*R* = 0.000), also revealed by the data computed for the M–M b.c.p.’s and by the Wiberg bond orders, summarized in Table 3. As for **2**, no b.c.p. was found between the carbonyl ligands and the coinage metal. The DFT-optimized geometry of **3**, including M–M b.c.p.’s, is shown in Figure 4. On the basis of the ρ and V data at b.c.p.’s, Ag(1)–Ru(1)/Ag(1)–Ru(6) bonds are stronger than those of Ag(1)–Ru(3)/Ag(1)–Ru(4) (see Scheme 2 for labeling). Accordingly, the Ag(1)–Ru(1)/Ag(1)–Ru(6) bond order is 0.412, while that of Ag(1)–Ru(3)/Ag(1)–Ru(4) is 0.354. The Ru–Ru bond strengths follow the order previously described for **2**, that is, Ru(2)–Ru(3)/Ru(4)–Ru(5) > Ru(1)–Ru(2)/Ru(5)–Ru(6) ≈ Ru(3)–Ru(4) > Ru(1)–Ru(3)/Ru(4)–Ru(6). The Ru(1)–Ru(3)/Ru(4)–Ru(6) bonds appear weaker in **3** with respect to **2**, while the AIM and Wiberg data related to the other Ru–Ru bonds do not show meaningful variations.

**Table 3:** Selected Computed Data (a.u.) at Metal–Metal b.c.p. for **3** (ρ = Electron Density; *V* = Potential Energy Density; *E* = Energy Density; ∇^2^ρ = Laplacian of Electron Density) and Wiberg Bond Orders

bond	ρ	*V*	*E*	∇^2^ρ	Wiberg b.o.
Ag(1)–Ru(1) = Ag(1)–Ru(6)	0.045	–0.039	–0.011	0.066	0.412
Ag(1)–Ru(3) = Ag(1)–Ru(4)	0.039	–0.037	–0.008	0.083	0.354
Ru(1)–Ru(2) = Ru(5)–Ru(6)	0.042	–0.029	–0.011	0.025	0.479
Ru(2)–Ru(3) = Ru(4)–Ru(5)	0.049	–0.037	–0.014	0.037	0.577
Ru(1)–Ru(3) = Ru(4)–Ru(6)	0.032	–0.022	–0.007	0.036	0.379
Ru(3)–Ru(4)	0.041	–0.027	–0.010	0.024	0.495

The molecular orbital energy levels computed at the PBEh-3c level are comparable for **2** and **3**, with the highest occupied molecular orbital (HOMO) and the lowest unoccupied molecular orbital (LUMO) gaps comprised between 4.3 and 4.5 eV (Figure S20 in the Supporting Information).

**Scheme 3:**
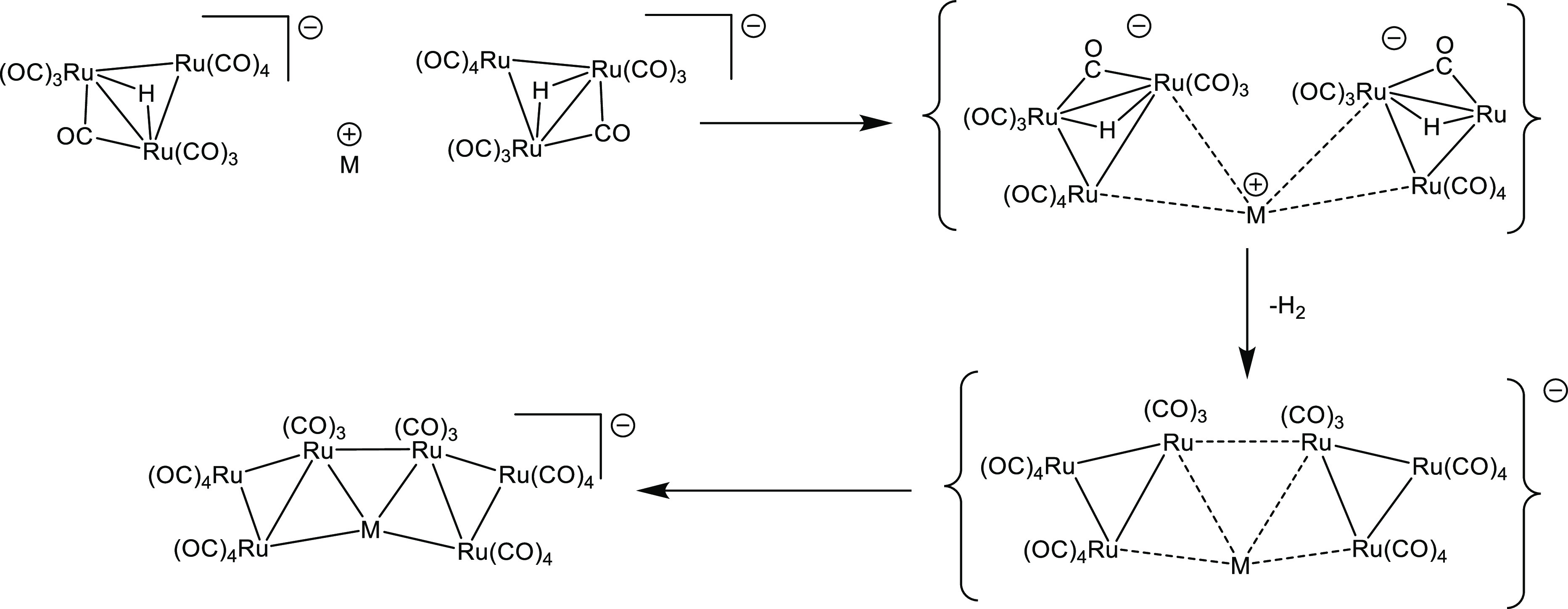
Proposed Mechanism for the Formation of [MRu_6_(CO)_22_]^−^

[MRu_6_(CO)_22_]^−^ may be viewed as composed of a [Ru_6_(CO)_22_]^2–^ anionic unit, which acts as a tetradentate ligand via four Ru atoms toward a single M^+^ cation. In view of this description, we can devise a possible formal mechanism for the formation of [MRu_6_(CO)_22_]^−^ (Scheme 3). First, two [HRu_3_(CO)_11_]^−^ units start to interact with a M^+^ ion. This interaction, somehow, promotes the oxidation of Ru clusters to [Ru_6_(CO)_22_]^2–^ with the concomitant reduction of H^+^ to H_2_, the formation of a direct inter-triangular Ru–Ru bond, and the rearrangement of the CO ligands. The direct involvement of the M^+^ ions in this process is corroborated by the fact that in their absence, the oxidation of [HRu_3_(CO)_11_]^−^ to [Ru_6_(CO)_22_]^2–^ is not observed.

**Figure 5:**
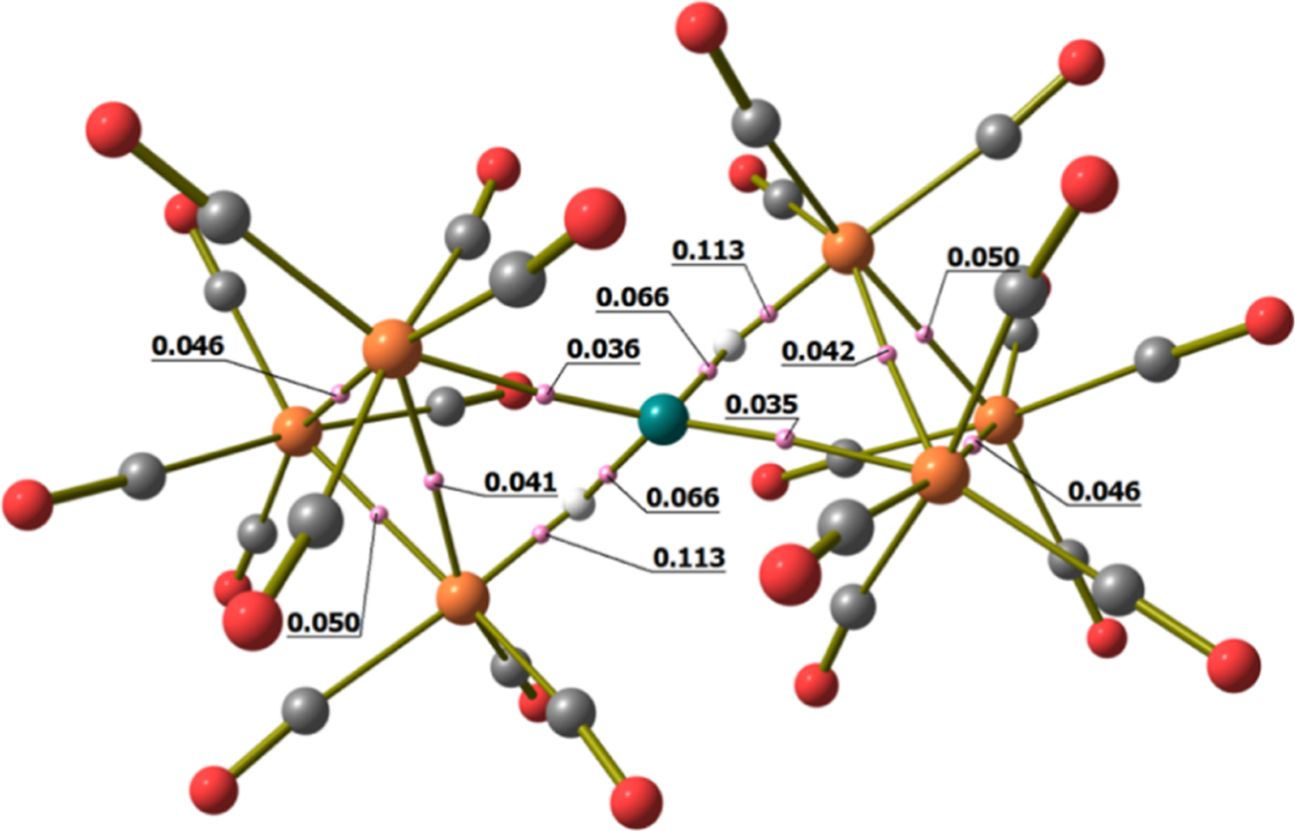
DFT-optimized structure of [Cu(μ-H)_2_{Ru_3_(CO)_11_}_2_]^−^ (orange Ru; green Cu; red O; gray C; white H) with M–M and M–H b.c.p.’s and corresponding ρ values (pink, a.u.).

Possible intermediate species involved in the formation of **2** from **1** were computationally investigated. In particular, the interaction of two **1** clusters with a copper monocation afforded the compound [Cu(μ-H)_2_{Ru_3_(CO)_11_}_2_]^−^ depicted in Figure 5 as the most stable stationary point. [Cu(μ-H)_2_{Ru_3_(CO)_11_}_2_]^−^ has an approximate *C*_2_ symmetry (*R* = 0.047). The trinuclear {Ru_3_} fragments maintain the triangular arrangement, and both the hydrides bridge the Cu and one of the Ru centers. Two Cu–Ru bonds are also observable, and their presence was confirmed by the AIM and Wiberg analyses. The computed energy variation (sum of electronic energy and nuclear repulsion) for the reaction (1) is −11.8 kcal mol^–1^ (−13.7 kcal mol^–1^ including CH_2_Cl_2_ as continuous medium).
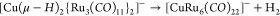
1

Electron density values at M–H and M–M b.c.p.’s are reported in Figure 5. Furthermore, AIM and Wiberg data are collected in Table S1 in the Supporting Information. The presence of Cu–H bonds was confirmed by all the computational analyses, but it is worth noting that the average Ru–H bond order is 0.556, much greater than the value related to the Cu–H interactions, 0.211.

### 2.2 Synthesis and Molecular Structure of [AuRu_5_(CO)_19_]^−^

As described in the previous section, **4** can be conveniently obtained from the reaction of **1** with Au(Et_2_S)Cl. [NEt_4_][AuCl_4_] may be employed as an alternative Au source.

**Figure 6:**
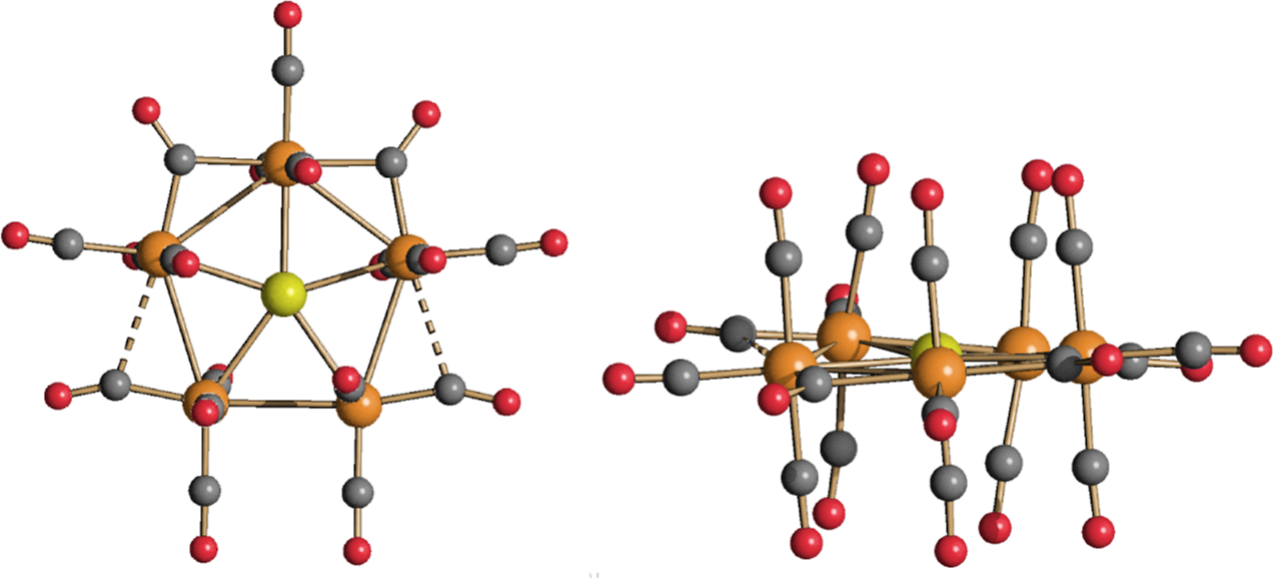
Molecular structure of [AuRu_5_(CO)_19_]^−^ (**4**) (orange Ru; yellow Au; red O; gray C). Au···C(O) contacts [2.81 Å] are represented as fragmented lines.

The solid-state structure of [NEt_4_][**4**] consists of an ionic packing of [NEt_4_]^+^ cations and **4** anions. The cluster anion (Figure 6 and Table 4) includes five Ru atoms, which compose a pentagon with rather elongated Ru–Ru edges [3.123(7)–3.184(4) Å]. The unique Au atom is at the center of the pentagon and forms five Au–Ru bonding contacts [2.664(3)–2.722(3) Å]. This results in an almost flat AuRu_5_ metal core [mean deviation from the AuRu_5_ least-square plane = 0.0781 Å] which is completed by 19 CO ligands, 15 terminals, and 2 edge bridging (α = 0.14) and 2 semi-bridging ligands (α = 0.51) on Ru–Ru bonds. In agreement with this, the IR spectrum of **4** recorded in CH_2_Cl_2_ displays v_CO_ bands both in the terminal [2049(m), 2022(vs), 2005(sh) cm^–1^] and bridging regions [1795(ms) cm^–1^].

**Table 4:** Main Bond Distances (Å) and Angles (°) of **4**^a^

Au(1)–Ru(1)	2.664(3)	Au(1)–Ru(2)	2.669(2)
	(1.04)		(1.04)
Au(1)–Ru(3)	2.722(3)	Ru(1)–Ru(2)	3.167(3)
	*(1.06)*		*(1.28)*
Ru(2)–Ru(3)	3.184(4)	Ru(3)–Ru(3A)	3.123(7)
	*(1.28)*		*(1.26)*
Ru–CO_terminal_^range^	1.79(2)–1.97(5)	Ru–CO_terminal_^average^	1.91(8)
Ru(1)–C(1)	2.23(3)	Ru(2)–C(1)	1.95(3)
Ru(2)–C(2)	2.81(4)	Ru(3)–C(2)	1.86(4)
sum angles at Au(1)	360.55(13)	Ru(2)–Ru(1)–Ru(2A)	107.27(9)
Ru(1)–Ru(2)–Ru(3)	107.96(9)	Ru(2)–Ru(3)–Ru(3A)	107.69(9)
Ru(1)–C(1)–O(1)	125(2)	Ru(2)–C(1)–O(1)	136(2)
Ru(3)–C(2)–O(2)	156(3)	Ru(2)–C(2)–O(2)	120(3)
α_CO(1)_	0.14	α_CO(2)_	0.51
mean deviation from AuRu_5_ least-square plane	0.0781		

a Cotton’s FSRs are reported in parentheses. Pauling’s atomic radii are employed: Ru 1.241 Å and Au 1.336 Å. See Scheme 4 for labeling.

As for **2** and **3**, the RMSD of the DFT-optimized structure with respect to X-ray data is small (0.280 Å). The AIM analysis was unable to locate b.c.p.’s between most of the ruthenium centers, with the exception of the Ru(3)–Ru(3A) bond (see Scheme 4 for labeling). On the contrary, b.c.p.’s were found for all the Ru–Au bonds. Data are summarized in Table 5. No b.c.p. was found for the weak Ru···CO contacts [Ru(2)···C(2) and Ru(2A)···C(2A)]. On the other hand, b.c.p.’s were localized for all the Ru–(μ-CO) bonds [Ru(1)–C(1), Ru(2)–C(1), Ru(1)–C(1A), and Ru(2A)–C(2A)]. The poor localization of the Ru–Ru b.c.p. is probably attributable to computational limits since the Wiberg analysis afforded bond orders between 0.3 and 0.4 for all the Ru–Ru bonds (Table 5). The strength order of the Ru–Au bonds, according to the data reported in Table 5, is Au(1)–Ru(1) ≈ Au(1)–Ru(2)/Au(1)–Ru(2A) > Au(1)–Ru(3)/Au(1)–Ru(3A). It is, however, worth noting that the ρ and V values are quite similar for all the Ru–Au b.c.p., and the bond orders are roughly comparable.

**Scheme 4:**
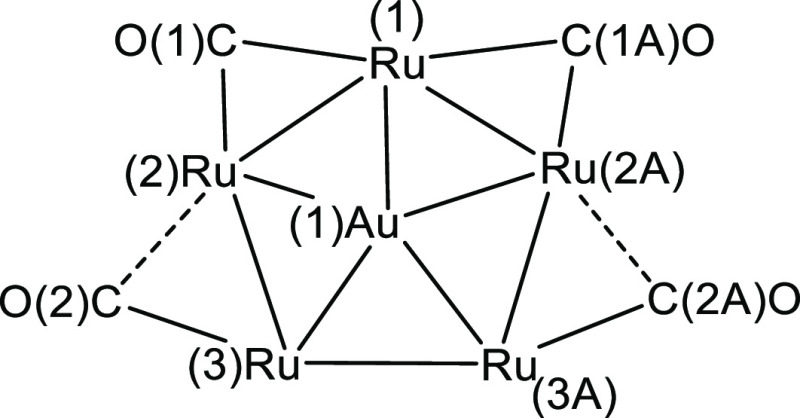
Labeling of **4**

**Table 5:** Selected Computed Data (a.u.) at Metal–Metal b.c.p.’s for **4** (ρ = Electron Density; *V* = Potential Energy Density; *E* = Energy Density; and ∇^2^ρ = Laplacian of Electron Density) and Wiberg Bond Orders

bond	ρ	*V*	*E*	∇^2^ρ	Wiberg b.o.
Au(1)–Ru(3)/Au(1)–Ru(3A)	0.053	–0.051	–0.013	0.099	0.445
Au(1)–Ru(1)	0.056	–0.054	–0.015	0.098	0.465
Au(1)–Ru(2)/Au(1)–Ru(2A)	0.056	–0.057	–0.014	0.114	0.470
Ru(3)–Ru(3A)	0.033	–0.019	–0.008	0.015	0.397
Ru(1)–Ru(2)/Ru(1)–Ru(2A)					0.352
Ru(2)–Ru(3)/Ru(2A)–Ru(3A)					0.307

The computed molecular orbital diagram (Figure S21 in the Supporting Information) does not highlight the presence of high-lying occupied orbitals, and the HOMO energy is close to the values obtained for **2** and **3**. The HOMO–LUMO gap is 4.50 eV, comparable with the values reported for the [MRu_6_(CO)_22_]^−^ (M = Cu and Ag) derivatives.

**Figure 7:**
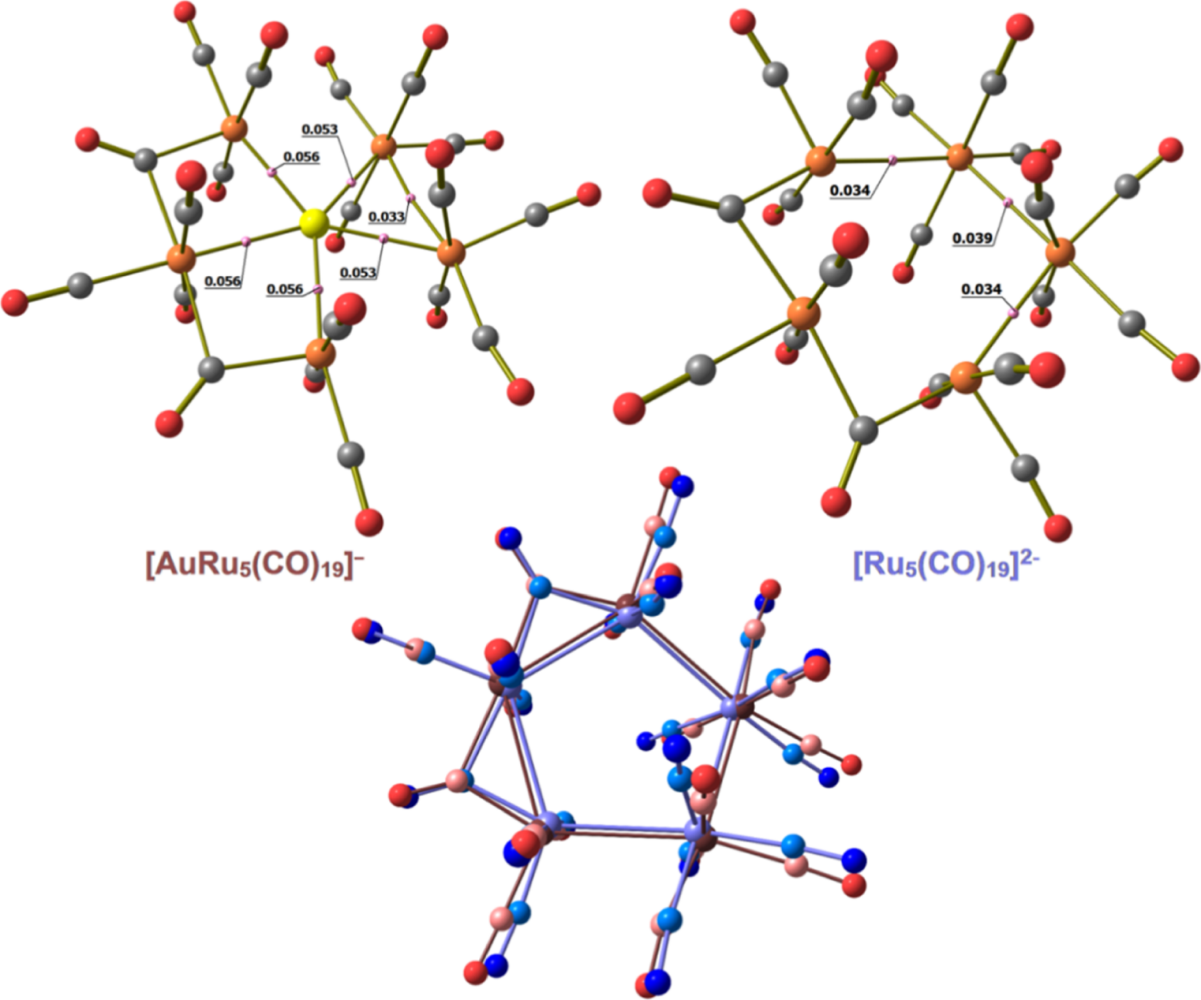
DFT-optimized structures of **4** and [Ru_5_(CO)_19_]^2–^ (orange Ru; yellow Au; red O; gray C) with M–M b.c.p.’s and corresponding ρ values (pink, a.u.) and the superposition of the {Ru_5_(CO)_19_} fragments of **4** (red tones) and [Ru_5_(CO)_19_]^2–^ (blue tones).

**Figure 8:**
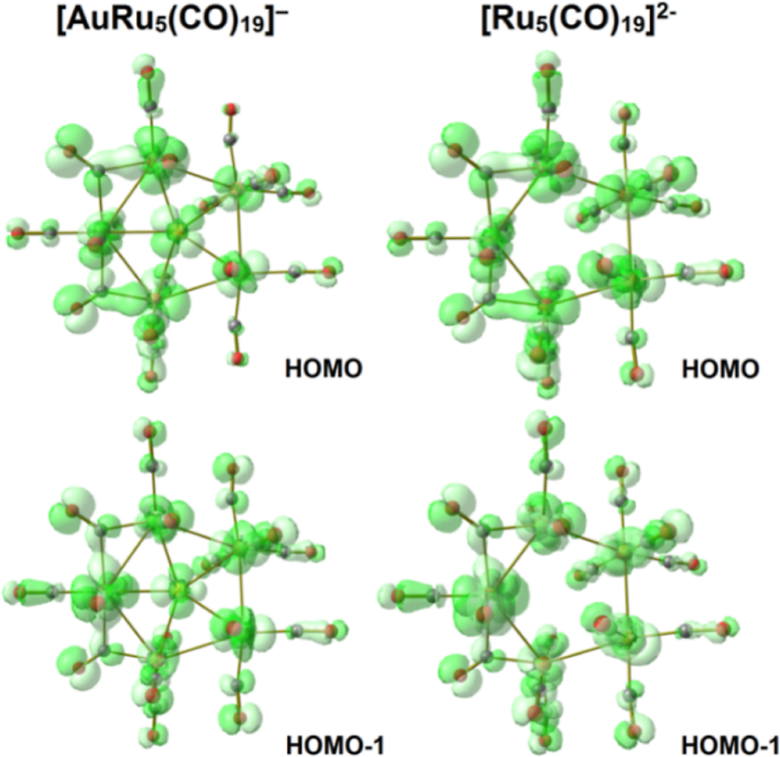
Selected molecular orbitals (green tones) of **4** and [Ru_5_(CO)_19_]^2–^. Surface isovalue = 0.03 a.u.

Compound **4** may be viewed as a Au(I) complex with the [Ru_5_(CO)_19_]^2–^ cluster which acts as a pentadentate ligand. The DFT-optimized structure of [Ru_5_(CO)_19_]^2–^ shows a pentagonal arrangement of the ruthenium centers comparable to that found for **4** (Figure 7, RMSD between the {Ru_5_(CO)_19_} fragments of 0.348 Å). The AIM data are different with respect to **4**, with an enforcement of the Ru(3)–Ru(3A) bond and the localization of b.c.p.’s between Ru(3) and Ru(2) and between Ru(3A) and Ru(2A). More importantly, the Ru–Ru Wiberg bond orders are composed of values between 0.41 and 0.49, meaningfully higher than those computed for **4** (Table 6). The Wiberg analysis therefore suggests that the formation of the Ru–Au bonds causes a weakening of the Ru–Ru interactions. The average Hirshfeld charge of the ruthenium centers is, however, roughly the same in both the compounds (0.05 a.u. in **4** and 0.07 a.u. in [Ru_5_(CO)_19_]^2–^), and analogies can be observed between the frontier occupied orbitals of **4** and [Ru_5_(CO)_19_]^2–^ (Figure 8), but the orbital energy values of [Ru_5_(CO)_19_]^2–^ are meaningfully higher, most likely because of the more negative charge of the compound, as observable in Figure S21 in the Supporting Information.

**Table 6:** Selected Computed Data (a.u.) at Metal–Metal b.c.p. for [Ru_5_(CO)_19_]^2–^ (ρ = Electron Density; *V* = Potential Energy Density; *E* = Energy Density; ∇^2^ρ = Laplacian of Electron Density) and Wiberg Bond Orders

bond	ρ	*V*	*E*	∇^2^ρ	Wiberg b.o.
Ru(3)–Ru(3A)	0.039	–0.024	–0.010	0.014	0.487
Ru(3)–Ru(2)/Ru(3A)–Ru(2A)	0.034	–0.023	–0.008	0.028	0.414
Ru(1)–Ru(2)/Ru(1)–Ru(2A)					0.464

### 2.3 Reactions of [MRu_6_(CO)_22_]^−^ (M = Cu and Ag) and [AuRu_5_(CO)_19_]^−^ with PPh_3_

**Scheme 5:**
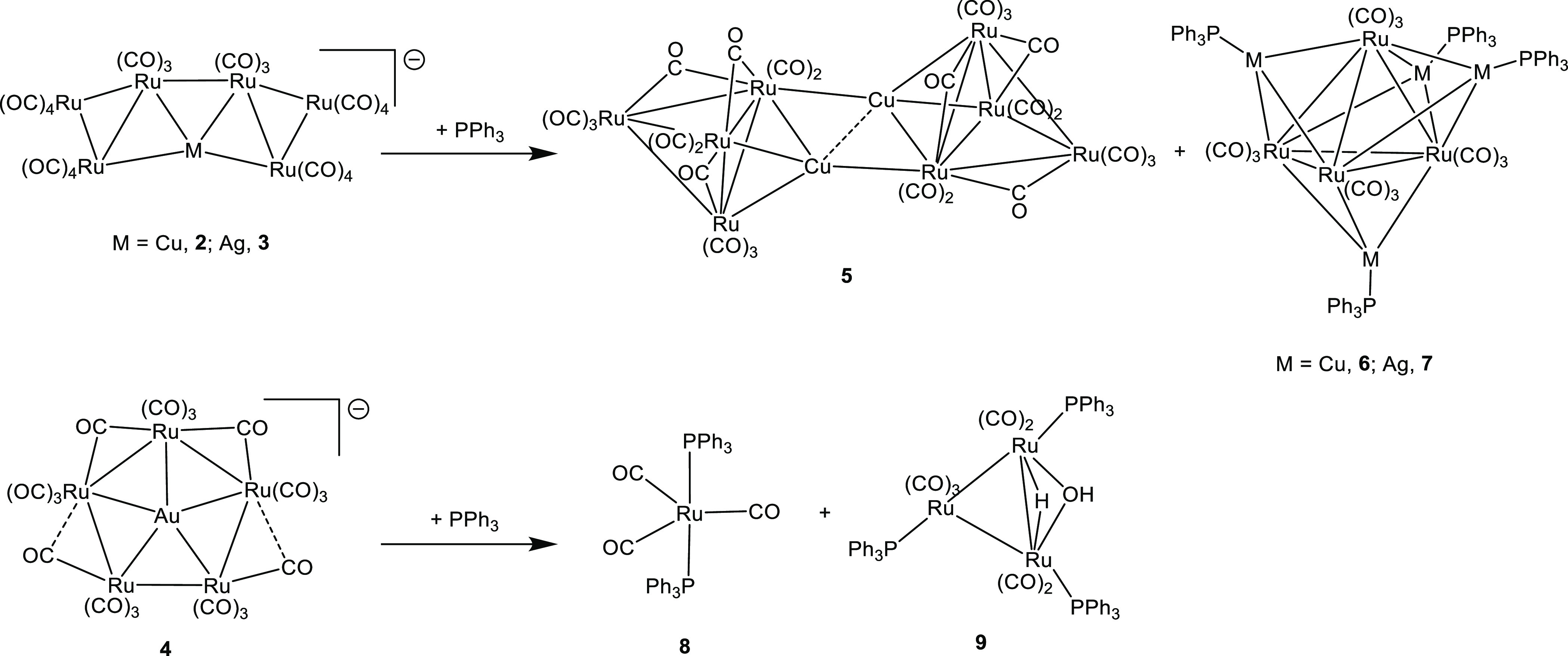
Reactions of **2–4** with PPh_3_

The reaction of **2** with PPh_3_ results in the formation of [Cu_2_Ru_8_(CO)_26_]^2–^ (**5**) and Ru_4_(CO)_12_(CuPPh_3_)_4_ (**6**) (Scheme 5). The two compounds can be separated owing to their different solubilities in the organic solvent. Thus, **6** can be extracted in toluene, whereas **5** as a [NEt_4_]^+^ salt is soluble in CH_2_Cl_2_. These two new compounds have been characterized by IR spectroscopy, and their molecular structures have been determined by SC-XRD as Ru_4_(CO)_12_(CuPPh_3_)_4_·solv (**6**·solv) and [NEt_4_]_2_[Cu_2_Ru_8_(CO)_26_]·1.5CH_2_Cl_2_ ([NEt_4_]_2_[**5**]·1.5CH_2_Cl_2_).

**Figure 9:**
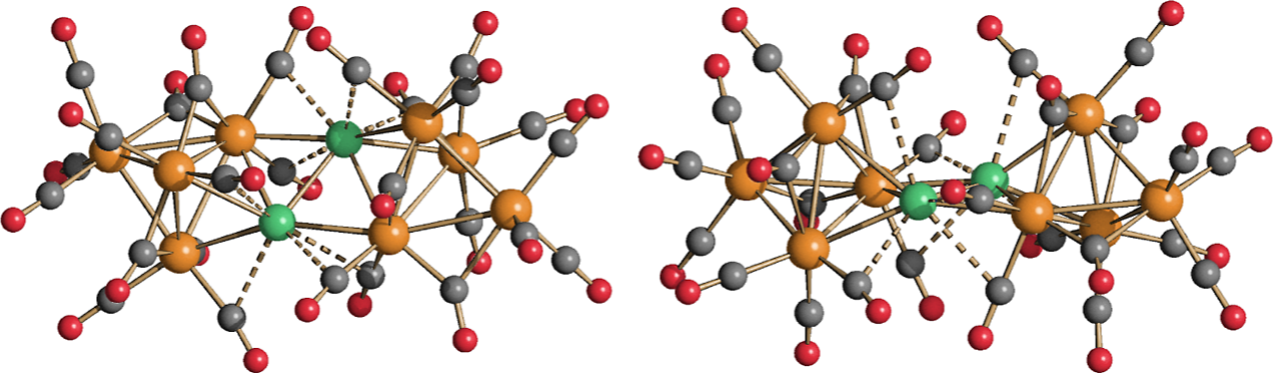
Molecular structure of [Cu_2_Ru_8_(CO)_26_]^2–^ (**5**) (orange Ru; green Cu; red O; gray C). Main bond distances (Å): Ru–Ru 2.7622(18)–2.9389(18), average 2.830(6); Ru–Cu 2.583(2)–2.731(2), average 2.654(6); Cu–Cu 2.514(3). Cu···C(O) contacts [2.33–2.74 Å] are represented as fragmented lines.

The anion **5** (Figure 9) may be viewed as composed of two tetrahedral [Ru_4_(CO)_13_]^2–^ units capped by two Cu^+^ cations. Each Cu^+^ cation is bonded to a triangular face of one [Ru_4_(CO)_13_]^2–^ tetrahedron and to a single Ru atom of the second [Ru_4_(CO)_13_]^2–^, belonging to the Ru_3_ triangle capped by the second Cu^+^. A Cu···Cu cuprophilic interaction [2.514(3) Å] is present.^42^ Each [Ru_4_(CO)_13_]^2–^ unit contains 10 terminal and three μ-CO ligands on Ru–Ru edges (α = 0.11–0.19). Some weak Cu···C(O) contacts [2.33–2.74 Å] are present (α = 0.23–0.44).

**Figure 10:**
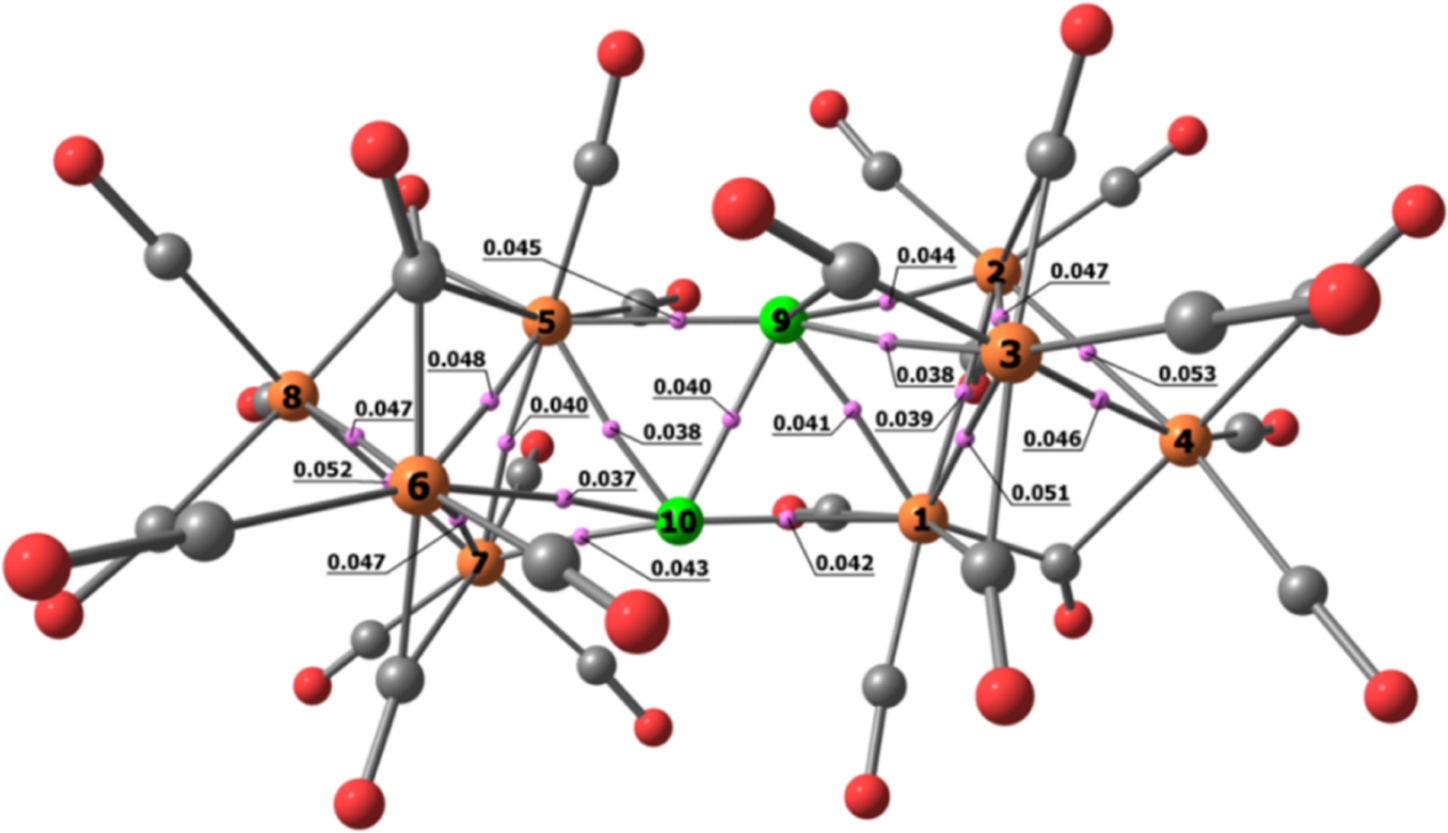
DFT-optimized structure of **5** (orange Ru; green Cu; red O; gray C) with M–M b.c.p.’s and corresponding ρ values (pink, a.u.).

The AIM analysis on the DFT-optimized structure of **5** (RMSD deviation with respect to the X-ray data equal to 0.404 Å) revealed the presence of (3,–1) b.c.p.’s in line with M–M bonds (see Figure 10 and Table S2 in the Supporting Information for selected AIM data). In particular, one b.c.p. between the two Cu atoms was localized. Four Cu–Ru b.c.p.’s are present for each copper center, three with one {Ru_4_} tetrahedron and one with the other {Ru_4_}. Five Ru–Ru b.c.p.’s were localized instead of the six expected for a Ru_4_ tetrahedron. In particular, the software was unable to find (3,–1) b.c.p.’s between Ru(1) and Ru(4) and between Ru(5) and Ru(8), as observable in Figure 10. All the expected bonds were instead found using the Wiberg analysis, with a Cu–Cu bond order of 0.194, Cu–Ru bond orders between 0.245 and 0.337, and Ru–Ru bond orders between 0.398 and 0.575.

**Figure 11:**
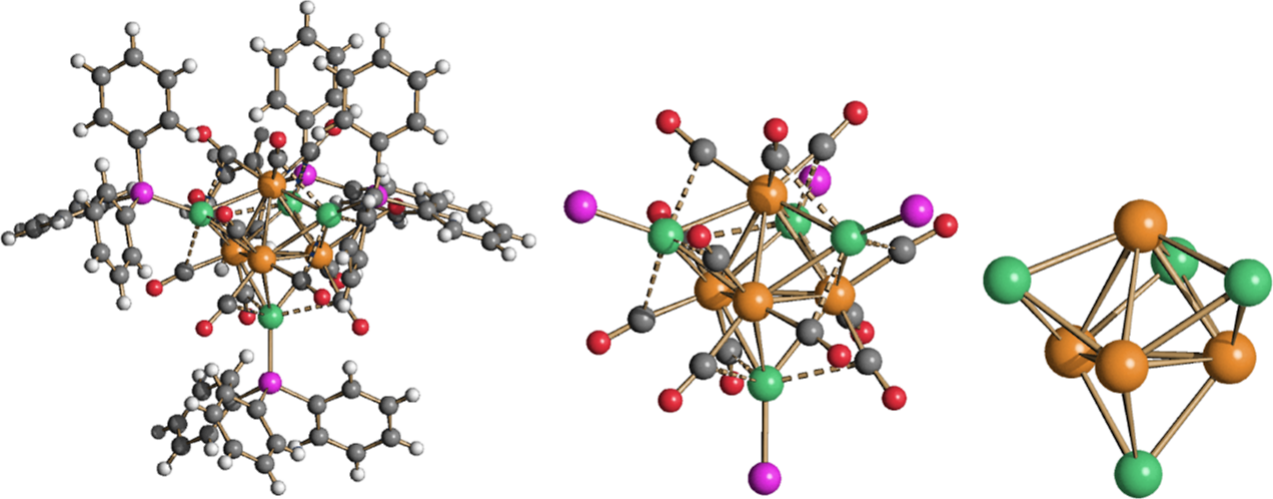
Molecular structure of Ru_4_(CO)_12_(CuPPh_3_)_4_ (**6**) (orange Ru; green Cu; red O; gray C). Main bond distances (Å): Ru–Ru 2.8570(6)–2.9021(11), average 2.8715(19); Ru–Cu 2.6247(8)–2.6725(10), and average 2.638(3). Cu···C(O) contacts [2.48–2.49 Å] are represented as fragmented lines.

Compound **6** is composed of a Ru_4_ tetrahedron whose four triangular faces are capped by four CuPPh_3_ groups (Figure 11). The CO ligands are all terminal, three per Ru atom, even though some weak Cu···C(O) contacts are present [2.48–2.49 Å; α = 0.31–0.32]. Based on the isolobal analogy between [CuPPh_3_]^+^ and H^+^,^43^**6** is related to H_4_Ru_4_(CO)_12_,^44^ even though the hydride ligands are edge bridging, whereas the Cu(I) fragments are face capping.

The reaction of **3** with PPh_3_ is very similar to that described above in the case of **2**. In particular, a neutral product is extracted at the end of the reaction in toluene, which shows an IR spectrum very similar to **6**. Thus, the product has been tentatively formulated as Ru_4_(CO)_12_(AgPPh_3_)_4_ (**7**).

**Figure 12:**
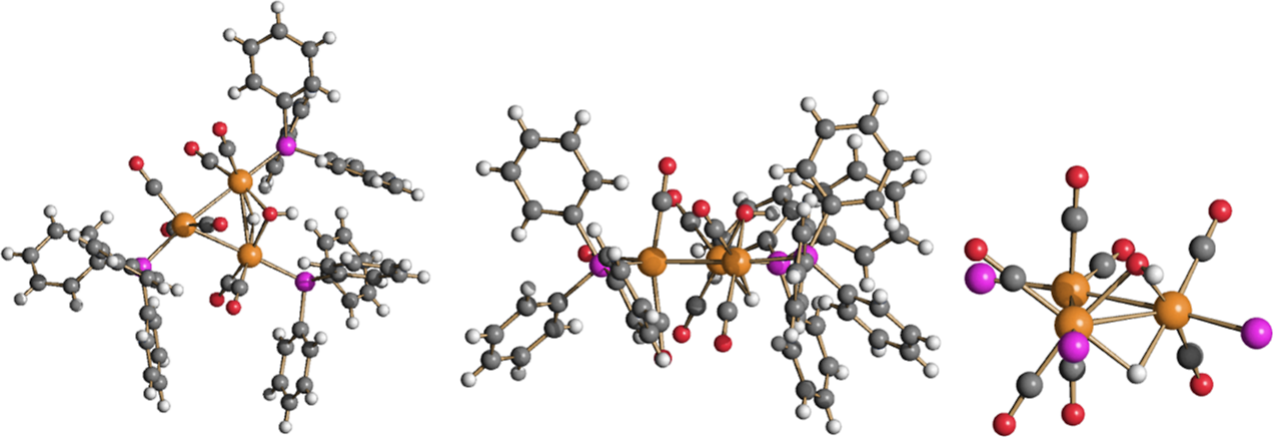
Molecular structure of HRu_3_(OH)(CO)_7_(PPh_3_)_3_ (**9**) (orange Ru; purple P; red O; gray C; white H).

Compound **4** reacts only with an excess of PPh_3_, affording mixtures of Ru(CO)_3_(PPh_3_)_2_ (**8**) and HRu_3_(OH)(CO)_7_(PPh_3_)_3_ (**9**). The yields of **9** may be improved after addition of some water to the reaction mixture. This is in keeping with the fact that the hydride and hydroxide ligands originate from H_2_O dissociation into H^+^ and OH^–^. The molecular structure of **9** has been ascertained by SC-XRD (Figure 12). It consists of a Ru_3_ triangle where one Ru–Ru edge is bridged by μ-H and μ-OH ligands. All the CO ligands are terminal and, in addition, there is one PPh_3_ ligand on each Ru atom.

The contemporary presence of μ-H and μ-OH ligands on the same M–M edge has been previously found in other dimers and clusters, such as HOs_3_(OH)(CO)_10_, HOs_3_(OH)(CO)_8_(PPh_3_)_2_, HOs_3_(OH)(CO)_8_(dppm), and HOs_3_(OH)(CO)_9_{SMe(Bu^*t*^)}.^45−48^

The ^31^P{^1^H} NMR spectrum of **9** in CD_2_Cl_2_ displays a sharp singlet at all temperatures (δ_P_ = 55.1 ppm), indicating a fast fluxional behavior (Figure S9 in the Supporting Information). This makes the three PPh_3_ ligands equivalent and, indeed, the μ-H hydride ligand appears as a quartet (*J*_H–P_ = 4 Hz) at δ_H_ −10.15 ppm in the ^1^H NMR spectrum (Figure S8 in the Supporting Information).

**Figure 13:**
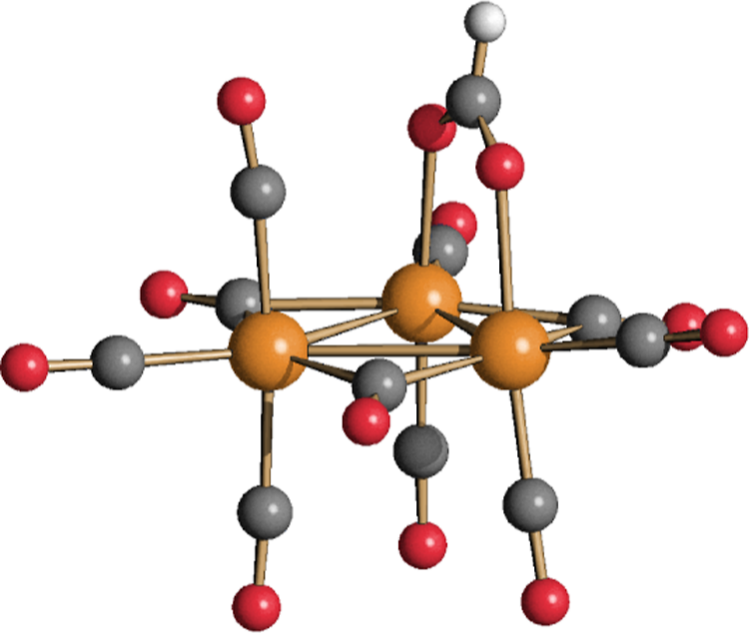
Molecular structure of [Ru_3_(CO)_10_(HCO_2_)]^−^ (**10**) (orange Ru; red O; gray C; white H).

As a final remark, in the attempt to test other Ag(I) reagents for the synthesis of Ru–Ag clusters, the reaction of **1** with Ag(PPh_3_)(NO_3_) has been investigated. This leads to **3** as the major product. Nonetheless, a few crystals of [NEt_4_][Ru_3_(CO)_10_(HCO_2_)] ([NEt_4_][**10**]) suitable for SC-XRD were obtained as a by-product of the reaction. The compound was obtained in very low yields and, therefore, it has only been possible to characterize it by SC-XRD (Figure 13). The structure is rather interesting since it consists of a Ru_3_ triangle, with three edge bridging carbonyls, seven terminal CO ligands, and one edge bridging formate ligand, HCO_2_^–^.^49,50^

### 2.4 Catalytic Tests

The [NEt_4_]^+^ salts of **2**, **3,** and **4** have been tested as catalyst precursors for transfer hydrogenation using ^*i*^PrOH as a solvent and a hydrogen source (Table 7). The model substrate employed was 4-fluoroacetophenone, and the reaction was monitored by ^19^F NMR spectroscopy. Since 4-F-α-methylbenzylalcohol [1-(4-fluorophenyl)ethan-1-ol] was the only product observed, only conversion was analyzed. The catalytic tests were performed employing 1 or 2.5% mol of catalyst precursor per mol of the substrate at a ^*i*^PrOH refluxing temperature (82 °C). Tests were carried out both in the absence and in the presence of a base (KO^*t*^Bu, 10% mol/mol with respect to the substrate). All catalytic tests have been carried out at least three times using different cluster catalyst precursor batches (including crystalline batches), resulting in highly reproducible results. This seems to exclude that what is being seen is catalysis by trace impurities.

**Table 7:**
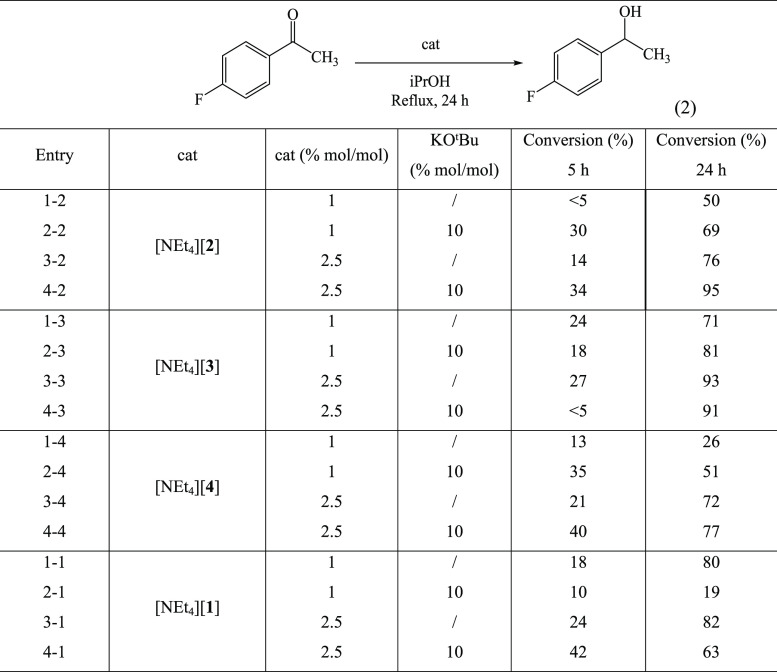
Catalytic Transfer Hydrogenation of 4-Fluoroacetophenone with Heterometallic [NEt_4_][**2**], [NEt_4_][**3**], and [NEt_4_][**4**] as Compared to Homometallic [NEt_4_][**1**]^a^

a General conditions: catalyst (3 or 7.5 μmol, 1% or 2.5% mol/mol), ^*i*^PrOH (5 mL), KO^*t*^Bu (10 mol % when added), and 4-fluoroacetophenone (36.5 μL, 300 μmol), *T* = 82 °C, N_2_ atmosphere; the conversions were determined by ^19^F NMR spectroscopy. All entries are the average of three independent catalytic runs.

The conversion after 24 h was observed in the range of 26–95%, suggesting some catalytic activity under all the experimental conditions considered. The conversion after 5 h was considerably lower (5–40%), indicating a long induction period. Such a long period is likely to be required in order to transform the catalyst precursors **2–4** into the active species. As described below, spectroscopic (IR, ^1^H NMR, and ESI-MS) analyses performed on the reaction mixtures at the end of the catalytic processes clearly indicate that mixtures of carbonyl clusters, including hydride carbonyl clusters, are present, ruling out cluster breakdown to mononuclear complexes or nanoparticles.

As expected, conversion increased by increasing the catalyst load for all three clusters, both in the absence and presence of the base. In the case of **2**, the addition of the base significantly increased the conversion at both catalyst loads. In contrast, in the case of **3** and **4**, the positive effect of base addition was significant at 1% mol/mol catalyst load, whereas it was almost negligible with a higher catalyst load. The activity as a catalyst precursor decreases in the order of **3** > **2** > **4**. Also, the effect of the base on the conversion after 5 h is different for the three clusters. In particular, in the case of the best catalyst precursor **3**, addition of the base has a negligible (or even negative effect) on the conversion after 5 h. This further corroborates the opinion that activation does not involve cluster breakdown but cluster transformation.

For comparison, homometallic cluster **1** was employed as a catalyst precursor under similar experimental conditions. The conversions measured for **1** in the absence of a base were rather good, whereas addition of the base had a strong detrimental effect, at a difference from **2–4**. The effect of increasing the catalyst load of **1** from 1 to 2.5% in the absence of the base had a negligible effect, also in this case, showing a different behavior as compared to **2–4**. The fact that the effects of the catalyst load and addition of the base were very different in the case of **2–4** and **1** suggests a different mechanism for the activation and/or catalysis in the case of heterometallic Ru–M clusters as compared to the homometallic one. Even if it is not possible at the moment to depict a mechanism, there is spectroscopic evidence of the fact that cluster breakdown to mononuclear species or nanoparticles does not occur both for heterometallic and homometallic precursors.

Control experiments have been carried out in order to check for potential background reactions under the experimental conditions adopted for the catalytic tests (Table S3 in the Supporting Information). These include reactions without any catalyst (with and without base) as well as reactions using simple Ru, Cu, Ag, or Au salts as potential catalyst precursors. The conversions after 5 and 24 h were almost zero for all these control experiments, pointing out that the results summarized in Table 7 are not affected by any background reaction. Moreover, since under the experimental conditions employed herein, no conversion is observed using simple M(I) salts (M = Cu, Ag, and Au) as potential catalyst precursors, it is likely to be excluded that, in the case of heterometallic clusters, the catalytic activity is somehow related to the formation of M(I) compounds resulting from cluster breakdown. This further corroborates the opinion that catalysis should be obtained after cluster activation and not cluster decomposition.

In order to test the possibility of reusing the catalyst, it was at first attempted to again add some substrate at the end of the first catalytic run, but the conversion was rather low (see entries 1-2-R and 3-2-R in Table 8). This might be due to decomposition and/or a negative effect of the reaction product. Indeed, by performing the catalysis starting from a 1:1 mixture of the substrate (4-fluoroacetophenone) and product (4-F-α-methylbenzylalcohol), the conversion after 24 h was rather lowered as compared to the same experiment in the absence of 4-F-α-methylbenzylalcohol (compare entries 1-2 and 1-2-P in Tables 7 and 8). In order to remove the negative effect of the product, the reaction mixture was dried under reduced pressure at the end of the catalytic run, and the residue was washed with *n*-hexane. Then, the solvent and the substrate were added again, and a second catalytic run was carried out, resulting in 0% conversion (entry 1-2-R–H in Table 8), probably because of the decomposition of the catalyst during the work-up, even if the system had been kept under an inert atmosphere during all the manipulations.

**Table 8:** Supplementary Tests on Catalytic Transfer Hydrogenation of 4-Fluoroacetophenone with [NEt_4_][**2**]^a^

entry	cat	cat (% mol/mol)	conversion (%) 24 h
1-2-P^b^	[NEt_4_][**2**]	1	13
1-2-R^c^		1	6
3-2-R^d^		2.5	10
1-2-R–H^e^		1	0

a General conditions as in Table 7. No base was added.

b 4-F-α-methylbenzyl alcohol (38 μL, 300 μmol) was added to the initial mixture under the same conditions of run 1-2; average of two catalytic runs.

c At the end of a run under the same conditions of 1-2 (24 h), 4-fluoroacetophenone (36.5 μL, 300 μmol) was added again, and the reaction was monitored after 24 h; single catalytic run.

d At the end of a run under the same conditions of 3-2 (24 h), 4-fluoroacetophenone (36.5 μL, 300 μmol) was added again, and the reaction was monitored after 24 h; single catalytic run.

e At the end of a run under the same conditions of 1–2 (24 h), the solvent was removed under reduced pressure, the residue was washed with *n*-hexane, 4-fluoroacetophenone (36.5 μL, 300 μmol), and ^*i*^PrOH (5 mL) were added again, and the reaction was monitored after 24 h; average of two catalytic runs.

The nature of the carbonyl species present in the reaction mixture at the end of the catalytic tests was investigated by combined IR, ^1^H NMR, and ESI-MS analyses. In addition, clusters **2–4** were heated in ^*i*^PrOH at a refluxing temperature, and the resulting products were spectroscopically analyzed. In all cases, complex mixtures of products were detected, including carbonyl hydride clusters. Among the different species, it was possible to identify [H_3_Ru_4_(CO)_12_]^−^ (**11**)^51,52^ and [HRu_6_(CO)_18_]^−^ (**12**).^53^ Other unidentified species were also present, probably also including heterometallic clusters. The fact that **2–4** were not present at the end of the catalytic tests indicated that they were transformed during catalysis. At the same time, it is possible to exclude that complete decomposition of the clusters to mononuclear species or metal nanoparticles occurs since cluster carbonyl species and hydride carbonyl clusters are still present, as indicated by IR, ^1^H NMR, and ESI-MS (Figures S10–S19 in the Supporting Information). Thus, it seems that the catalyst precursors **2–4** are transformed into the active species during the induction period. The fact that mixtures of clusters are present at the end of the catalytic process (with no evidence of mononuclear species) suggests that cluster breakdown does not occur during the whole catalytic process. Therefore, this should involve only cluster transformations from precursors to active species and, eventually, to inactive species.

The formation of such complex mixtures of products and, in particular, the presence of hydrides are likely to be due to the use of a protic solvent such as ^*i*^PrOH. Indeed, the thermal treatment under mild conditions (60–80 °C) of **2–4** in aprotic solvents such as tetrahydrofuran (THF) or CH_3_CN resulted in the elimination of the coinage metal and formation of [Ru_6_(CO)_18_]^2–^ (**13**). The nature of this previously reported cluster^54^ has been deduced from IR data and further corroborated by SC-XRD analyses on the new salts [NEt_4_]_2_[**13**]·CH_2_Cl_2_ and [NEt_4_]_2_[**13**]·CH_3_COCH_3_ (Figure S7 in the Supporting Information). It is well known that **13** under protic conditions is transformed into **12**.^53^

Compound **13** resulted in 40% conversion after 24 h at 1% catalyst load, showing inferior performances to **1–3** under the same experimental conditions. This would suggest that the activation of heterometallic clusters **2** and **3** does not involve the formation of **13**.

## 3 Conclusions

Three new heterometallic Ru–M (M = Cu, Ag, and Au) carbonyl clusters **2–4** possessing a quasi-planar metal core have been fully characterized. They add to the limited but very interesting class of 2-D molecular alloy clusters. The coinage metal within **2–4** formally retains the +1 oxidation state and, thus, they may be viewed as M(I) complexes containing the organometallic ligands η^4^-[Ru_6_(CO)_22_]^2–^ and η^5^-[Ru_5_(CO)_19_]^2–^. The formal apticity of the polynuclear ruthenium anions in their interactions with the coinage metals was confirmed by AIM and Wiberg analyses on DFT-optimized structures. The computed data support the idea that the Ru–M bonds give major contributions to the stabilization of **2–4**. Clusters **2–4** displayed some activity as catalyst precursors in the transfer hydrogenation of 4-fluoroacetophenone. The fact that catalyst load and addition of a base had a different effect on **2–4** as compared to homometallic cluster **1** suggested that their heterometallic nature is somehow involved in the catalytic process.

## 4 Experimental Section

### 4.1 General Procedures

All reactions and sample manipulations were carried out using standard Schlenk techniques under nitrogen and in dried solvents. All the reagents were commercial products (Aldrich) of the highest purity available and used as received, except [NEt_4_][HRu_3_(CO)_11_] ([NEt_4_][**1**]), which had been prepared according to the literature.^52^ Analyses of C, H, and N were obtained with a ThermoQuest Flash EA 1112NC instrument. IR spectra were recorded on a PerkinElmer Spectrum One interferometer in CaF_2_ cells. ^1^H, ^13^C{^1^H}, ^19^F{^1^H}, and ^31^P{^1^H} NMR measurements were performed on a Varian Mercury Plus 400 MHz instrument. The proton and carbon chemical shifts were referenced to the non-deuterated aliquot of the solvent. The fluorine chemical shifts were referenced to external CCl_3_F. The phosphorus chemical shifts were referenced to external H_3_PO_4_ (85% in D_2_O). Structure drawings have been performed with SCHAKAL99.^55^

### 4.2 Synthesis of [NEt_4_][CuRu_6_(CO)_22_] ([NEt_4_][**2**])

[Cu(CH_3_CN)_4_][BF_4_] (0.065 g, 0.208 mmol) was added slowly as a solid to a solution of [NEt_4_][**1**] (0.300 g, 0.404 mmol) in CH_2_Cl_2_ (15 mL). The resulting mixture was stirred at room temperature for 1 h. Then, the solvent was removed under reduced pressure, and the residue was washed with water (40 mL) and toluene (40 mL) and extracted with CH_2_Cl_2_ (10 mL). The red CH_2_Cl_2_ solution was layered with *n*-pentane (30 mL), affording crystals of [NEt_4_][**2**] suitable for SC-XRD (yield 0.223 g, 78% based on Ru and 76% based on Cu).
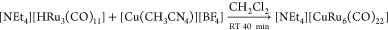
3

C_30_H_20_CuNO_22_Ru_6_ (1416.43): calcd (%): C, 25.44; H, 1.42; N, 0.99. Found: C, 25.71; H, 1.20; N, 0.78. IR (CH_2_Cl_2_, 298 K) ν_CO_: 2069(ms), 2037(ms), 2013(vs), 1946(sh) cm^–1^. IR (Nujol, 298 K) ν_CO_: 2064(m), 1998(s), 1959(w), 1938(w) cm^–1^. ATR–FTIR (298 K) ν_CO_: 2058(w), 1989(s), 1973(s), 1953(s), 1934(s), 1809(w) cm^–1^.

### 4.3 Synthesis of [NEt_4_][AgRu_6_(CO)_22_] ([NEt_4_][**3**])

#### 4.3.1 From [NEt_4_][HRu_3_(CO)_11_] and AgNO_3_

AgNO_3_ (0.034 g, 0.202 mmol) was added slowly as a solid to a solution of [NEt_4_][**1**] (0.300 g, 0.404 mmol) in CH_2_Cl_2_ (15 mL). The resulting mixture was stirred at room temperature for 1 h. Then, the solvent was removed under reduced pressure, and the residue was washed with water (40 mL) and toluene (40 mL) and extracted with CH_2_Cl_2_ (10 mL). The red CH_2_Cl_2_ solution was layered with *n*-pentane (30 mL), affording crystals of [NEt_4_][**3**] suitable for SC-XRD (yield 0.206 g, 70% based on Ru and 70% based on Ag).
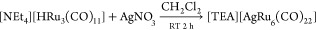
4

#### 4.3.2 From [NEt_4_][HRu_3_(CO)_11_] and Ag(Et_2_S)(NO_3_)

Ag(Et_2_S)(NO_3_) (0.038 g, 0.145 mmol) is added as a solid in three portions to a solution of [NEt_4_][**1**] (0.179 g, 0.241 mmol) in CH_2_Cl_2_ (15 mL) under a nitrogen atmosphere. The reaction required 1 h for each addition. Then, the solvent was removed under reduced pressure, and the residue was washed with water (40 mL) and toluene (40 mL). [NEt_4_][**3**] was extracted in CH_2_Cl_2_ (10 mL). The red CH_2_Cl_2_ solution was layered with *n*-pentane (30 mL), affording crystals of [NEt_4_][**3**] (yield 0.106 g, 60% based on Ru, 50% based on Ag).
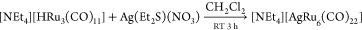
5

C_30_H_20_AgNO_22_Ru_6_ (1460.76): calcd (%): C, 24.67; H, 1.38; N, 0.96. Found: C, 24.33; H, 1.69; N, 1.14. IR (CH_2_Cl_2_, 298 K) ν_CO_: 2067(w), 2034(sh), 2022(sh), 2008(vs), 1969(m) cm^–1^. ATR–FTIR (298 K) ν_CO_: 2071(w), 2018(sh), 1997(s), 1964(m), 1931(w) cm^–1^.

### 4.4 Synthesis of [NEt_4_][AuRu_5_(CO)_19_] ([NEt_4_][**4**])

#### 4.4.1 From [NEt_4_][HRu_3_(CO)_11_] and Au(Et_2_S)Cl

Au(Et_2_S)Cl (0.060 g, 0.185 mmol) was added slowly as a solid to a solution of [NEt_4_][**1**] (0.300 g, 0.404 mmol) in CH_2_Cl_2_ (15 mL). The resulting mixture was stirred at room temperature for 1 h. Then, the solvent was removed under reduced pressure, and the residue was washed with water (40 mL) and toluene (40 mL) and extracted with CH_2_Cl_2_ (10 mL). The brown CH_2_Cl_2_ solution was layered with *n*-pentane (30 mL), affording crystals of [NEt_4_][**4**] suitable for SC-XRD (yield 0.179 g, 54% based on Ru, 71% based on Au).
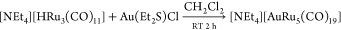
6

#### 4.4.2 From [NEt_4_][HRu_3_(CO)_11_] and [NEt_4_][AuCl_4_]

[NEt_4_][AuCl_4_] (0.031 g, 0.0653 mmol) was added as a solid in three portions to a solution of [NEt_4_][**1**] (0.149 g, 0.201 mmol) in CH_2_Cl_2_ (15 mL) under a nitrogen atmosphere. The reaction required 1 h for each addition. Then, the solvent was removed under reduced pressure, and the residue was washed with water (40 mL) and toluene (40 mL). [NEt_4_][**4**] was extracted in CH_2_Cl_2_ (10 mL). The brown CH_2_Cl_2_ solution was layered with *n*-pentane (30 mL), affording crystals of [NEt_4_][**4**] (yield 0.064 g, 39% based on Ru and 72% based on Au).
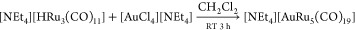
7

C_27_H_20_AuNO_19_Ru_5_ (1364.76): calcd (%): C, 23.76; H, 1.48; N, 1.03. Found: C, 23.45; H, 1.14; N, 0.79. IR (CH_2_Cl_2_, 298 K) ν_CO_: 2049(s), 2018(vs), 1988(ms), 1750(m) cm^–1^. IR (Nujol, 298 K) ν_CO_: 2037(m), 2006(ms), 1963(ms), 1927(m), 1723(w) cm^–1^. ATR–FTIR (298 K) ν_CO_: 2006(m), 1955(s), 1922(vs), 1764(m) cm^–1^.

### 4.5 Reactivity of [NEt_4_][CuRu_6_(CO)_22_] with PPh_3_: Synthesis of [NEt_4_]_2_[Cu_2_Ru_8_(CO)_26_] ([NEt_4_]_2_[5]) and Ru_4_(CO)_12_(CuPPh_3_)_4_·solv (**6**·solv)

PPh_3_ (0.145 g, 0.552 mmol) was added slowly as a solid to a solution of [NEt_4_][**2**] (0.260 g, 0.184 mmol) in CH_2_Cl_2_ (15 mL). The resulting mixture was stirred at room temperature for 6 h. Then, the solvent was removed under reduced pressure, and the residue was washed with water (40 mL) and extracted with toluene (10 mL). The toluene solution was layered with *n*-hexane, affording crystals of **6**·solv suitable for SC-XRD (yield 0.023 g, 4% based on Ru and 24% based on Cu). Then, the residue was extracted with CH_2_Cl_2_ (10 mL), and the red solution was layered with *n*-pentane, affording crystals of [NEt_4_]_2_[**5**]·1.5CH_2_Cl_2_ suitable for SC-XRD (yield 0.102 g, 36% based on Ru, 54% based on Cu).
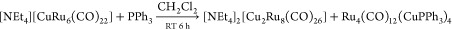
8

**6**·solv: C_84_H_60_Cu_4_O_12_P_4_Ru_4_ (2043.64): calcd (%): C, 49.37; H, 2.96. Found: C, 49.11; H, 3.15. IR (toluene, 298 K) ν_CO_: 1950(s), 1907(w) cm^–1^. IR (Nujol, 298 K) ν_CO_: 1950(s), 1907(m) ^31^P{^1^H} NMR (400 MHz, CD_2_Cl_2_, 298 K): δ_P_ 27.4 ppm.

[NEt_4_]_2_[**5**]: C_43.5_H_43_Cl_3_Cu_2_N_2_O_26_Ru_8_ (2051.79): calcd (%): C, 25.46; H, 2.11; N, 1.37. Found: C, 25.17; H, 1.86; N, 1.59. IR (CH_2_Cl_2_, 298 K) ν_CO_: 2042(w), 2001(sh), 1984(s), 1771(m) cm^–1^. IR (Nujol, 298 K) ν_CO_: 2047(m), 2023(m), 1976(s) cm^–1^.

### 4.6 Reactivity of [NEt_4_][AuRu_5_(CO)_19_] with PPh_3_

Solid PPh_3_ (0.201 g, 0.769 mmol) was added in small portions to a solution of [NEt_4_][**4**] (0.350 g, 0.256 mmol) in CH_2_Cl_2_ (20 mL); the mixture was stirred at room temperature, and the reaction was monitored by IR spectroscopy. At the end of the reaction, the solution was evaporated to dryness, and the residue was washed with water (40 mL) and hot EtOH (40 mL) and then extracted with toluene (15 mL). The toluene solution was layered with *n*-hexane, affording crystals of HRu_3_(OH)(CO)_7_(PPh_3_)_3_·1.5toluene (**9**·1.5toluene) suitable for SC-XRD as the main product (yield 0.101 g, 16% based on Ru) together with a few crystals of Ru(CO)_3_(PPh_3_)_2_ (**8**) as a by-product. Some Au metals remained on the reaction flask at the end of the reaction.
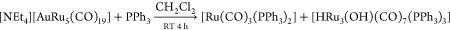
9

**9**·1.5toluene: C_71.5_H_59_O_8_P_3_Ru_3_ (1442.30): calcd (%): C, 59.54; H 4.12. Found: C, 59.77; H 3.86. IR (CH_2_Cl_2_, 298 K) ν_CO_: 2027(w), 1996(m), 1972(w), 1957(s), 1898(w) cm^–1^. IR (Nujol, 298 K) ν_CO_: 2024(w), 1992(m), 1972(w), 1954(s), 1930(m) cm^–1^. ^1^H NMR (CD_2_Cl_2_, 298 K): δ_H_ −10.15 (q, *J*_H–P_ = 4 Hz) ppm. ^31^P{^1^H} NMR (CD_2_Cl_2_, 298 K): δ 55.1 ppm.

**8**: IR (CH_2_Cl_2_, 298 K) ν_CO_: 1889 cm^–1^.

### 4.7 Reactivity of [NEt_4_][Ru_3_(CO)_11_] with Ag(PPh_3_)(NO_3_)

Ag(PPh_3_)(NO_3_) (0.014 g, 0.0324 mmol) was added as a solid in five portions to a solution of [NEt_4_][**1**] (0.744 g, 0.552 mmol) in CH_2_Cl_2_ (15 mL) under a nitrogen atmosphere. The reaction required 20 min for each addition. Then, to speed up the reaction, it was heated at 45 °C for 1 h. Then, the solvent was removed under reduced pressure, and the residue was washed with water (40 mL) and toluene (20 mL). The main product of the reaction, which is [NEt_4_][**3**], was extracted in CH_2_Cl_2_ (10 mL). However, by layering the CH_2_Cl_2_ solution with *n*-pentane, a few crystals of [NEt_4_][Ru_3_(CO)_10_(HCO_2_)] ([NEt_4_][**10**]) suitable for SC-XRD were obtained as a by-product of the reaction.
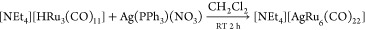
10

### 4.8 Reactivity of [NEt_4_][AgRu_6_(CO)_22_] with PPh_3_

PPh_3_ (0.074 g, 0.282 mmol) was added as a solid in small portions to a solution of [NEt_4_][**3**] (0.250 g, 0.188 mmol) in CH_2_Cl_2_ (15 mL). The resulting mixture was stirred at room temperature for 4 h. Then, the solvent was removed under reduced pressure, and the residue was washed with water (40 mL) and extracted with toluene (10 mL). The compound was formulated as Ru_4_(CO)_12_(AgPPh_3_)_4_ (**7**) by comparison of its IR spectrum with that of **6**.
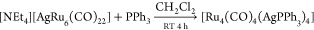
11

IR (toluene, 298 K) ν_CO_: 1949 and 1907(w) cm^–1^.

### 4.9 X-ray Crystallographic Study

Crystal data and collection details for [NEt_4_][**2**], [NEt_4_][**3**], [NEt_4_][**4**], [NEt_4_]_2_[**5**]·1.5CH_2_Cl_2_, **6**·solv, **9**·1.5toluene, [NEt_4_][**10**], [NEt_4_]_2_[**13**]·CH_2_Cl_2_, and [NEt_4_]_2_[**13**]·CH_3_COCH_3_ are reported in Table S4 in the Supporting Information. The diffraction experiments were carried out on a Bruker Apex II diffractometer equipped with a PHOTON2 detector using Mo Kα radiation. Data were corrected for Lorentz polarization and absorption effects (empirical absorption correction SADABS).^56^ Structures were solved by direct methods and refined by full-matrix least-squares based on all data using *F*^2^.^57^ Hydrogen atoms were fixed at calculated positions and refined with a riding model. All non-hydrogen atoms were refined with anisotropic displacement parameters unless otherwise stated.

### 4.10 Computational Details

Geometry optimizations were performed in the gas phase using the PBEh-3c method, which is a reparametrized version of PBE0^58^ (with 42% HF exchange) that uses a split-valence double-zeta basis set (def2-mSVP) with relativistic ECPs for Ru, Ag, and Au^59^ and adds three corrections that consider dispersion, basis set superposition, and other basis set incompleteness effects.^60^ Single point calculations with the addition of the C-PCM solvation model were also carried out, considering dichloromethane as continuous medium.^61^ The “restricted” approach was used in all the cases. Calculations were performed with ORCA 4.0.1.2.^62^ The output, converted in a .molden format, was elaborated with the software Multiwfn, version 3.5.^63^ The Cartesian coordinates of the DFT-optimized structures are provided in a separate .xyz file.

### 4.11 General Procedure for Transfer Hydrogenation Catalytic reactions

In a 10 mL two-neck round-bottom flask equipped with a condenser, cluster (3 or 7.5 μmol, 1 or 2.5% mol/mol) and KO^*t*^Bu (10 mol % when needed) were dissolved in ^*i*^PrOH (5 mL) and stirred at reflux temperature under a nitrogen atmosphere for 5 min. Then 4-fluoroacetophenone (36.5 μL, 300 μmol) was added, and samples were taken at regular intervals (1, 3, 5, and 24 h of reaction). Aliquots (100 μL) were diluted with CDCl_3_ (0.5 mL), and conversions were determined by ^19^F NMR spectroscopy.

### 4.12 Transfer Hydrogenation of 4-Fluoroacetophenone with [NEt_4_][**2**] in the Presence of an Internal Standard

In a 10 mL two-neck round-bottom flask equipped with a condenser, [NEt_4_][**2**] (7.5 μmol, 2.5% mol/mol) and ^*i*^PrOH (5 mL) were stirred at reflux temperature under a nitrogen atmosphere for 5 min. Then, the substrate 4-fluoroacetophenone (36.5 μL, 300 μmol) and α,α,α-trifluorotoluene (36.8 μL, 300 μmol), as internal standards, were added. After 24 h of reaction, a sample was taken and right after, 4-fluoroacetophenone (36.5 μL, 300 μmol) was added again. The last sample was taken at 48 h of reaction. Aliquots (100 μL) were diluted with CDCl_3_ (0.5 mL), and conversions were determined by ^19^F NMR spectroscopy.

### 4.13 Reactivity Experiment of [NEt_4_][**2**] in ^*i*^PrOH

In a 10 mL two-neck round-bottom flask equipped with a condenser, [NEt_4_][**2**] (90 mg, 64 μmol) and ^*i*^PrOH (7 mL) were stirred at reflux temperature under a nitrogen atmosphere, and then, 4-fluoroacetophenone (7.8 μL, 64 μmol) was added. The reaction mixture was stirred at reflux temperature under the nitrogen atmosphere. After 24 h, the solvent was removed under vacuum, and the crude of the reaction was analyzed by IR, ^1^H NMR, and ESI-MS.

### 4.14 Reactivity Experiment of [NEt_4_][**3**] in ^*i*^PrOH

In a 10 mL two-neck round-bottom flask equipped with a condenser, [NEt_4_][**3**] (45 mg, 31 μmol) and ^*i*^PrOH (7 mL) were stirred at reflux temperature under a nitrogen atmosphere, and then, 4-fluoroacetophenone (3.75 μL, 31 μmol) was added. The reaction mixture was stirred at reflux temperature under the nitrogen atmosphere. After 24 h, the solvent was removed under vacuum, and the crude of the reaction was analyzed by IR, ^1^H NMR, and ESI-MS.

### 4.15 Reactivity Experiment of [NEt_4_][**1**] in ^*i*^PrOH

In a 10 mL two-neck round-bottom flask equipped with a condenser, [NEt_4_][**1**] (74.5 mg, 100 μmol) and ^*i*^PrOH (7 mL) were stirred at reflux temperature under a nitrogen atmosphere, and then, 4-fluoroacetophenone (12.10 μL, 100 μmol) was added. The reaction mixture was stirred at reflux temperature under the nitrogen atmosphere. After 24 h, the solvent was removed under vacuum, and the crude of the reaction was analyzed by IR, ^1^H NMR, and ESI-MS.

### 4.16 Transfer Hydrogenation of 4-Fluoroacetophenone with [NEt_4_][**2**] in the Presence of 4-F-α-methylbenzylalcohol and an Internal Standard

In a 10 mL two-neck round-bottom flask equipped with a condenser, [NEt_4_][**2**] (3 μmol, 1% mol/mol) and ^*i*^PrOH (5 mL) were stirred at reflux temperature under a nitrogen atmosphere for 5 min. Then, the substrate 4-fluoroacetophenone (36.5 μL, 300 μmol) and α,α,α-trifluorotoluene (36.8 μL, 300 μmol), as internal standards, and 4-F-α-methylbenzylalcohol (38 μL, 300 μmol) were added. Samples were taken at regular intervals (1, 3, 5, and 24 h of reaction). Aliquots (100 μL) were diluted with CDCl_3_ (0.5 mL), and conversions were determined by ^19^F NMR spectroscopy.

### 4.17 Transfer Hydrogenation of 4-Fluoroacetophenone with [NEt_4_][**2**] with Recycling of the Catalytic Species

In a 10 mL two-neck round-bottom flask equipped with a condenser, [NEt_4_][**2**] (3 μmol, 1% mol/mol) and ^*i*^PrOH (5 mL) were stirred at reflux temperature under a nitrogen atmosphere for 5 min. Then, the substrate 4-fluoroacetophenone (36.5 μL, 300 μmol) was added. After 24 h of reaction, a sample was taken; the solvent was removed under vacuum, and the solid was washed with three 5 mL aliquots of hexane. Right after the removal of the solvent under vacuum, ^*i*^PrOH (5 mL) and 4-fluoroacetophenone (36.5 μL, 300 μmol) were added again, and the flask was put at reflux temperature under the nitrogen atmosphere. The last sample was taken at 48 h of reaction. Aliquots (100 μL) were diluted with CDCl_3_ (0.5 mL) and conversions were determined by 19F NMR spectroscopy.

### 4.18 Reactivity Experiment of [NEt_4_][**2**] in CH_3_CN

A solution of [NEt_4_][**2**] (0.171 g, 0.121 mmol) in CH_3_CN (15 mL) under a nitrogen atmosphere was heated at 80 °C for 6 h. Then, the solvent was removed under reduced pressure, and the residue was washed with water (40 mL) and toluene (20 mL), and the product was extracted with acetone (10 mL). Crystals of [NEt_4_]_2_[**12**]·CH_3_COCH_3_ were obtained by slow diffusion of *n*-hexane on the acetone solution.

### 4.19 Reactivity Experiment of [NEt_4_][**4**] in THF

A solution of [NEt_4_][**4**] (0.171 g, 0.125 mmol) in THF (15 mL) under a nitrogen atmosphere was heated at 66 °C for 12 h. Then, the solvent was removed under reduced pressure, the residue was washed with water (40 mL) and toluene (20 mL), and the product was extracted with CH_2_Cl_2_ (10 mL). Crystals of [NEt_4_]_2_[**12**]·CH_2_Cl_2_ were obtained by slow diffusion of *n*-hexane on the CH_2_Cl_2_ solution.

## Supporting Information Available

DFT-optimized coordinates in the XYZ format (XYZ)
Supplementary experimental and computational figures and tables and crystal data and collection details (PDF)

## Supplementary Material

ic2c02099_si_001.xyz
