# Supplementary material for: 2-D Molecular Alloy Ru–M (M = Cu, Ag, and Au) Carbonyl Clusters: Synthesis, Molecular Structure, Catalysis, and Computational Studies
Source: Inorg Chem. 2022 Sep 7;61(37):14726–41. doi: 10.1021/acs.inorgchem.2c02099 (PMC9490753; doi:10.1021/acs.inorgchem.2c02099)
Supplement: Supplementary file 2 — ic2c02099_si_002.pdf [file ic2c02099_si_002.pdf]

Supporting Information for

2-D Molecular Alloy Ru-M (M = Cu, Ag, Au) Carbonyl Clusters: Synthesis, Molecular Structure, Catalysis and Computational Studies

Cristiana Cesari,[a,b*] Marco Bortoluzzi,[c] Francesca Forti,[a,b] Lisa Gubbels,[a] Cristina Femoni,[a] Maria Carmela Iapalucci,[a] and Stefano Zacchini[a,b]

[a] Dipartimento di Chimica Industriale "Toso Montanari", Università di Bologna, Viale Risorgimento 4 - 40136 Bologna. Italy. E-mail: cristiana.cesari2@unibo.it

[b] Center for Chemical Catalysis – C3, Viale Risorgimento 4 - 40136, University of Bologna, Italy.

[c] Dipartimento di Scienze Molecolari e Nanosistemi, Ca' Foscari University of Venice, Via Torino 155 – 30175 Mestre (Ve), Italy.

	Page/s
IR spectra	S2-S4
Molecular structure of **13**	S5
NMR spectra	S6-S8
ESI-MS spectra	S9-S13
Supplementary computational tables and diagrams	S14-S15
Transfer hydrogenation - Table of Control Experiments	S16
X-Ray crystallographic study	S17-S20

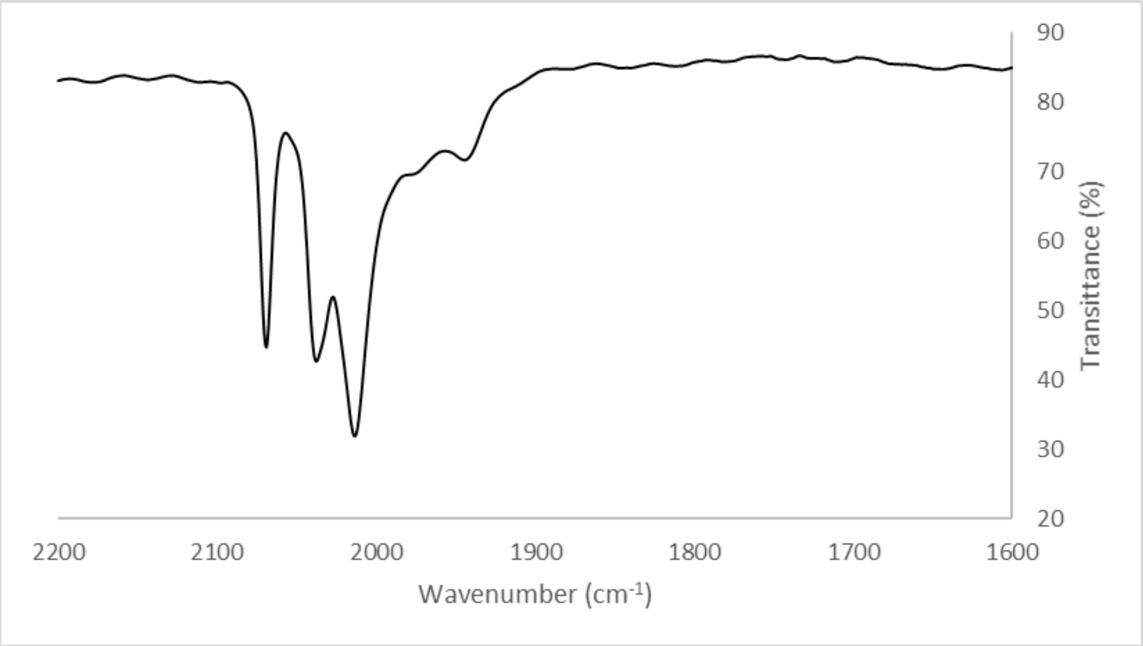

Figure S1. IR spectrum in the ν_{CO} region of $[NEt_4][CuRu_6(CO)_{22}]$ (**2**) in CH_2Cl_2.

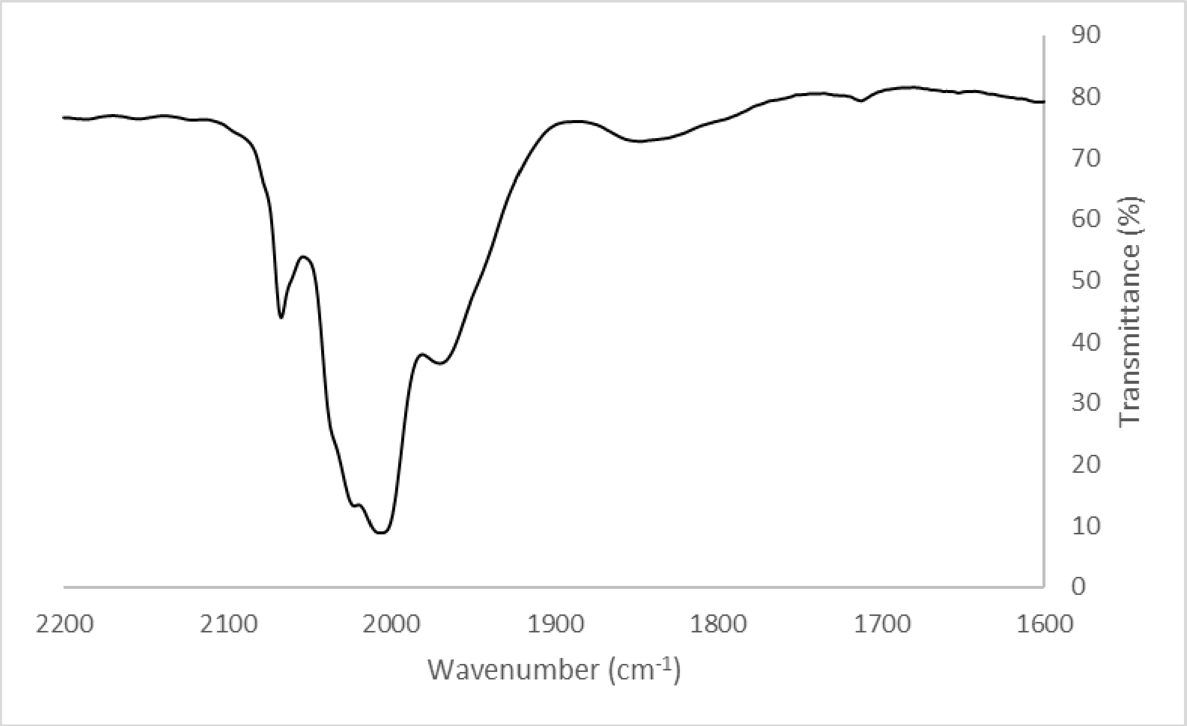

Figure S2. IR spectrum in the ν_{CO} region of $[NEt_4][AgRu_6(CO)_{22}]$ (**3**) in CH_2Cl_2.

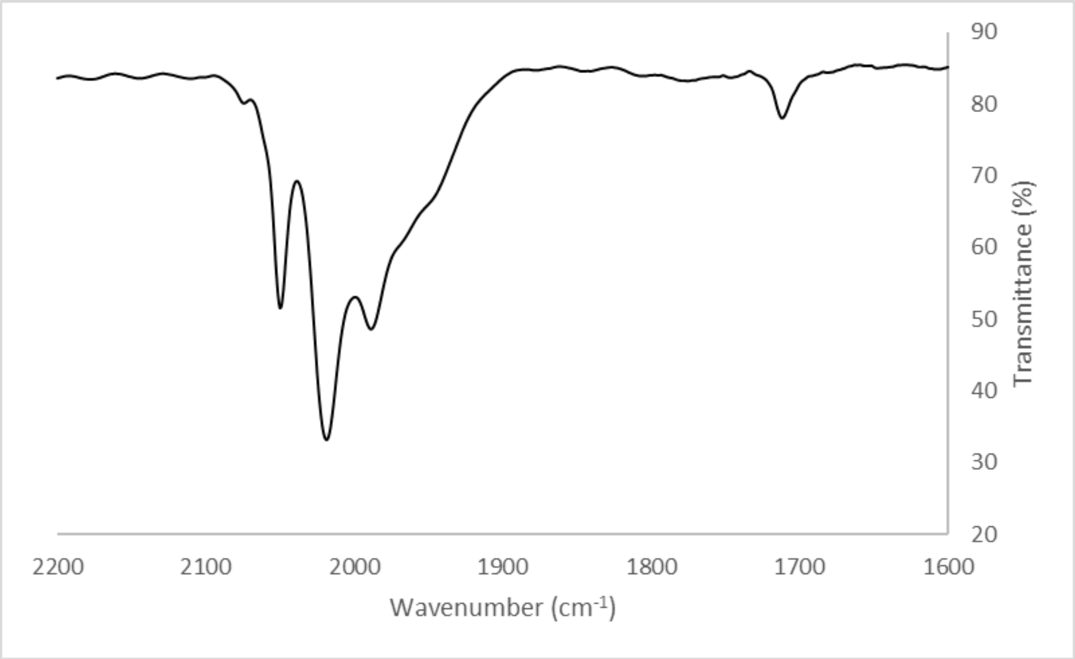

Figure S3. IR spectrum in the ν_{CO} region of $[NEt_4][AuRu_5(CO)_{19}]$ (**4**) in CH_2Cl_2.

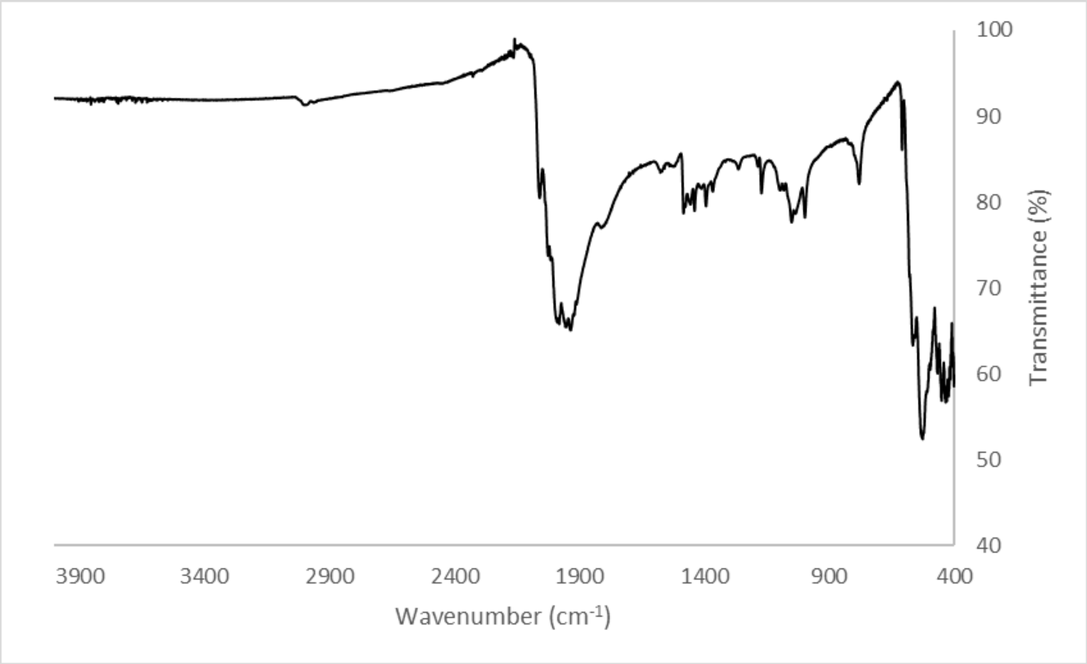

Figure S4. ATR-FTIR spectrum in the of $[NEt_4][CuRu_6(CO)_{22}]$ (**2**).

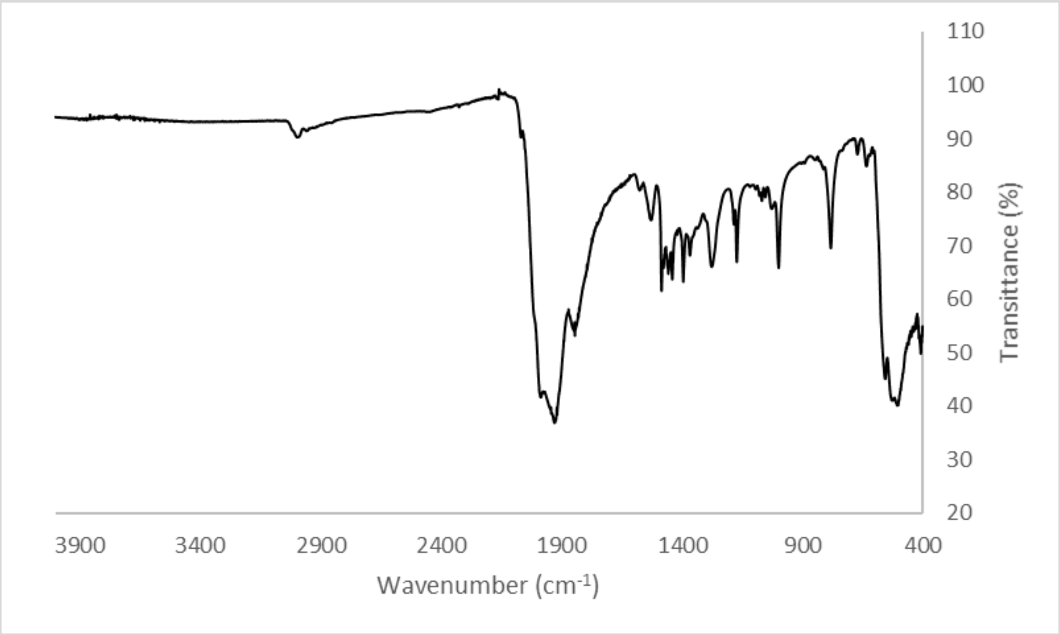

Figure S5. ATR-FTIR spectrum in the of $[NEt_4][AgRu_6(CO)_{22}]$ (**3**).

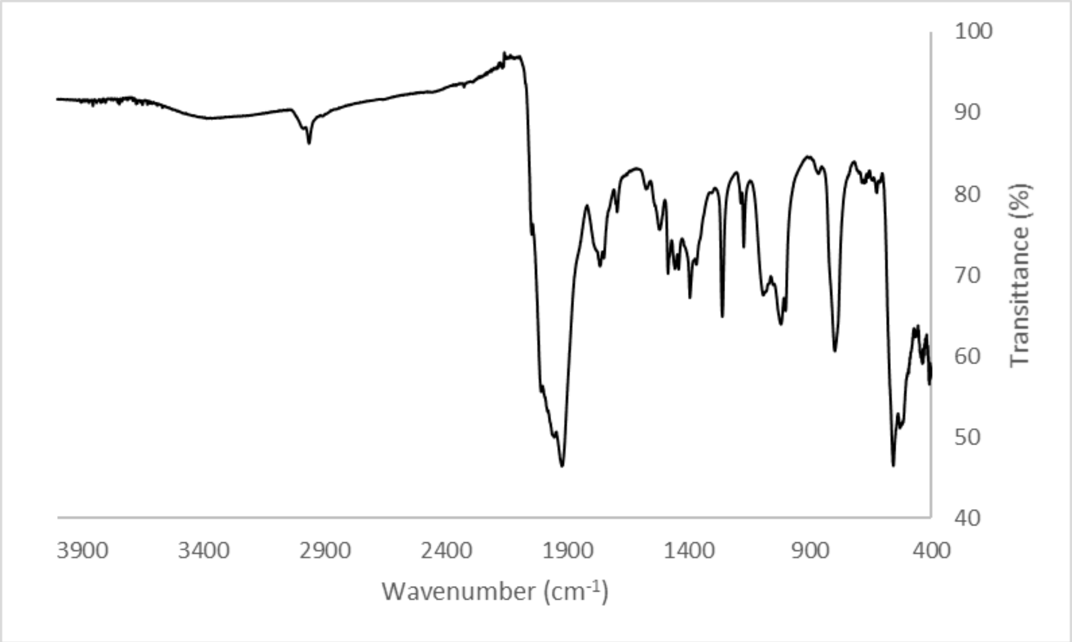

Figure S6. ATR-FTIR spectrum in the of$[NEt_4][AuRu_5(CO)_{19}]$ (**4**).

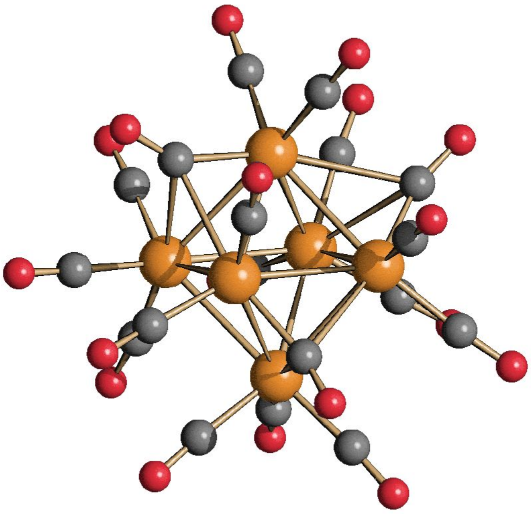

Figure S7. Molecular structure of $[Ru_6(CO)_{18}]^{2-}$ (**13**) (orange Ru; red O; grey C).

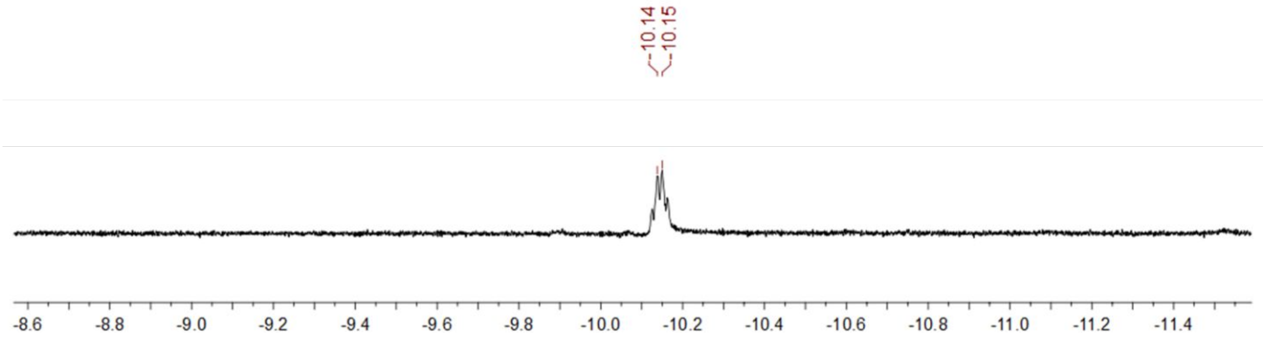

Figure S8. Hydride region of the ¹H NMR spectrum of $[HRu_3(OH)(CO)_7(PPh_3)_2]$ (**9**) in toluene-d^6 at 298 K.

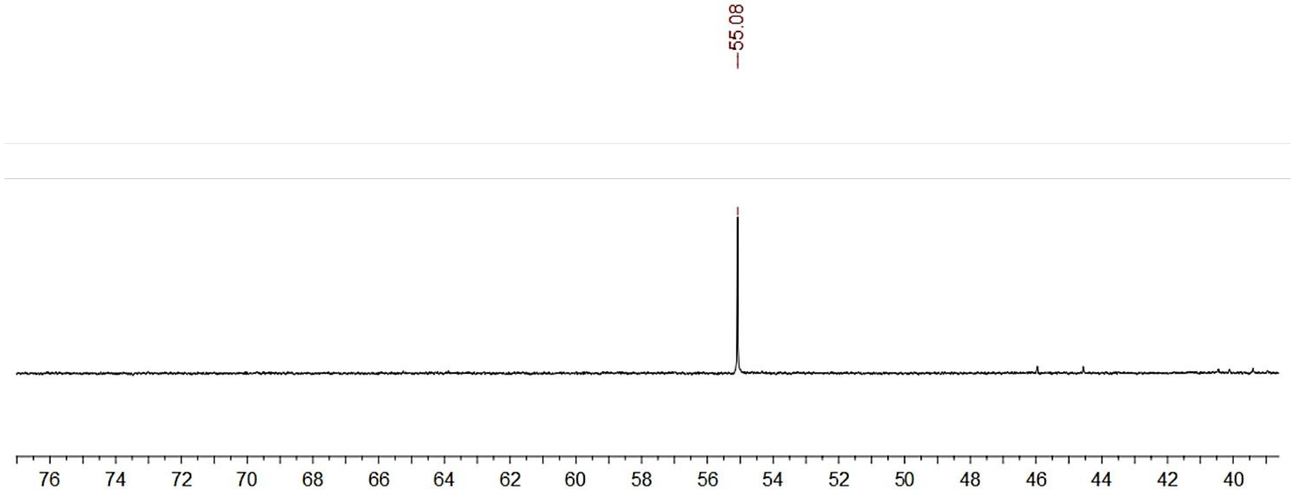

Figure S9. $^{31}P\{^1H\}$ NMR spectrum of $[HRu_3(OH)(CO)_7(PPh_3)_2]$ (**9**) in toluene-d^6 at 298 K.

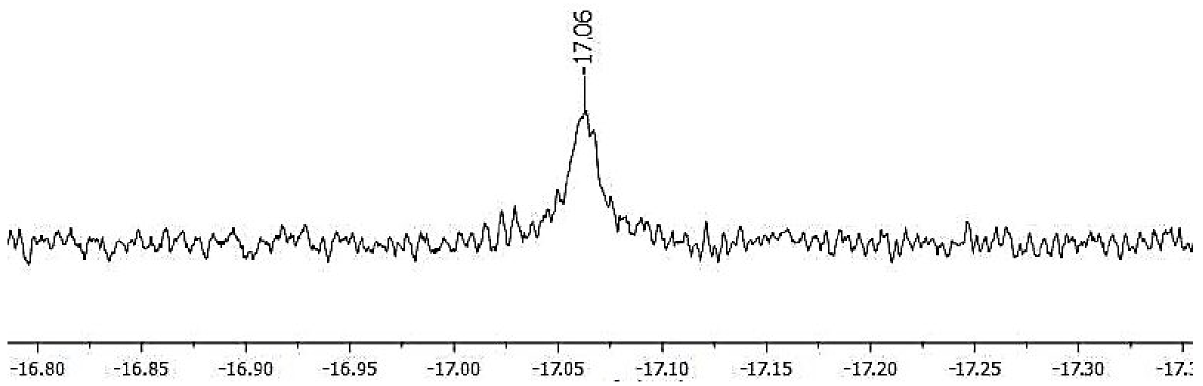

Figure S10. Hydride region of the ^{1}H NMR spectrum in acetone-d^6 at 298 K of the mixture at the end of the reactivity experiment: $[NEt_4][CuRu_6(CO)_{22}]$ (**2**) in ^{i}PrOH at 82 °C for 24 h.

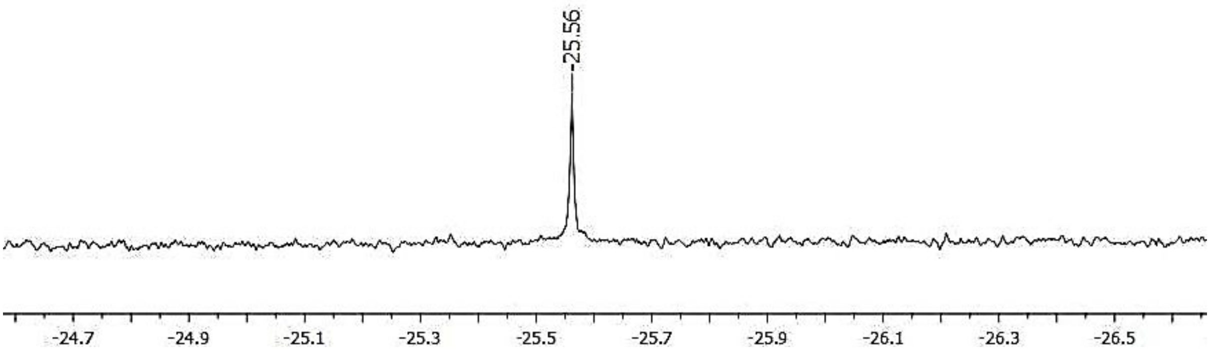

Figure S11. Hydride region of the ^{1}H NMR spectrum in acetone-d^6 at 298 K of the mixture at the end of the reactivity experiment: $[NEt_4][CuRu_6(CO)_{22}]$ (**2**) in ^{i}PrOH for 24 h at 82 °C, in the presence of substrate 4-Fluoroacetophenone, acetone extraction.

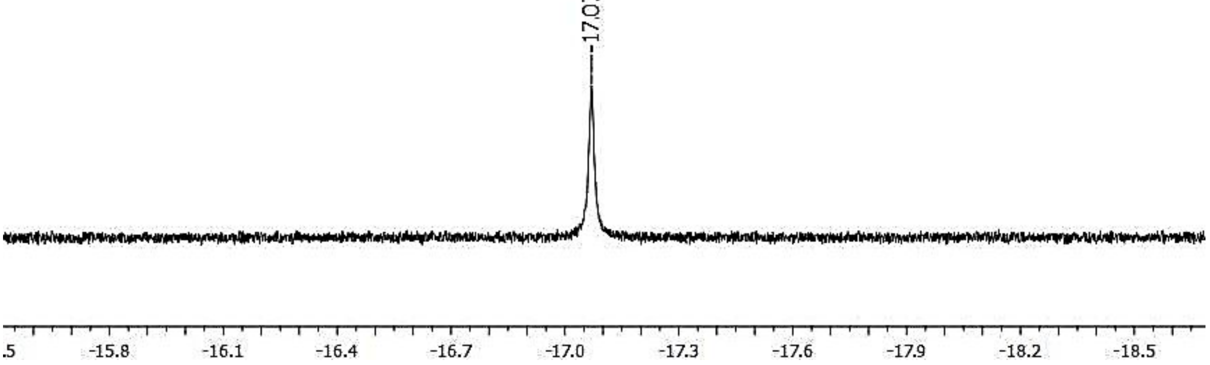

Figure S12. Hydride region of the ^{1}H NMR spectrum in CD_2Cl_2 at 298 K of the mixture at the end of the reactivity experiment: $[NEt_4][CuRu_6(CO)_{22}]$ (**2**) in ^{i}PrOH for 24 h at 82 °C, in the presence of substrate 4-Fluoroacetophenone, CH_2Cl_2 extraction.

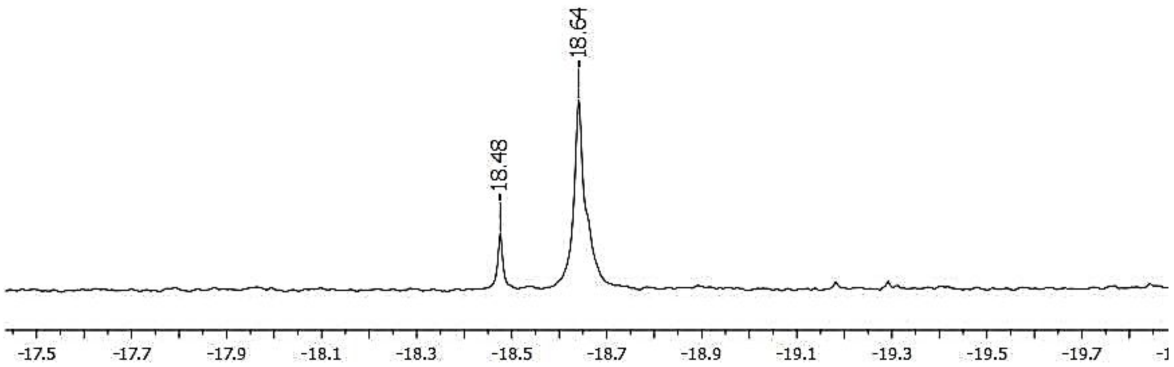

Figure S13. Hydride region of the ^{1}H NMR spectrum in acetone-d^{6} at 298 K of the mixture at the end of the reactivity experiment: $[NEt_4][AgRu_6(CO)_{22}]$ (**3**) in ^{i}PrOH for 24 h at 82 °C.

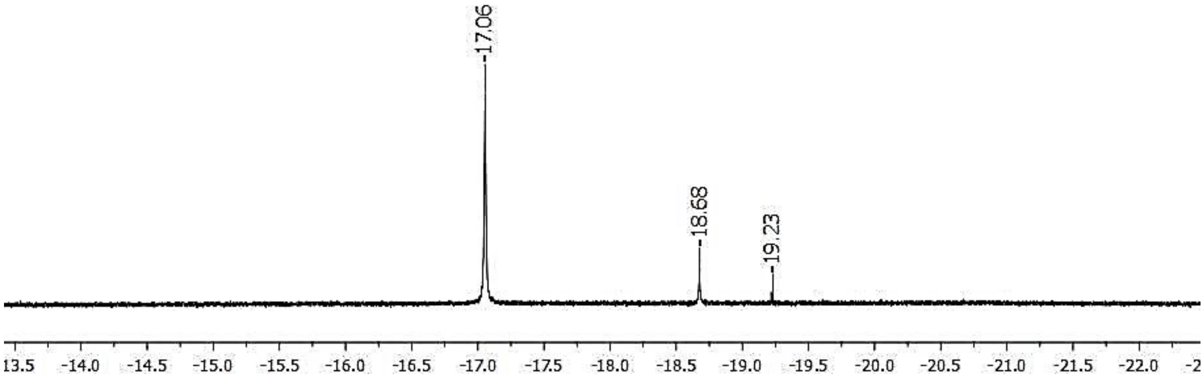

Figure S14. Hydride region of the ^{1}H NMR spectrum in acetone-d^{6} at 298 K of the mixture at the end of the reactivity experiment: $[NEt_4][AgRu_6(CO)_{22}]$ (**3**) in ^{i}PrOH for 24 h at 82 °C, in the presence of substrate 4-Fluoroacetophenone.

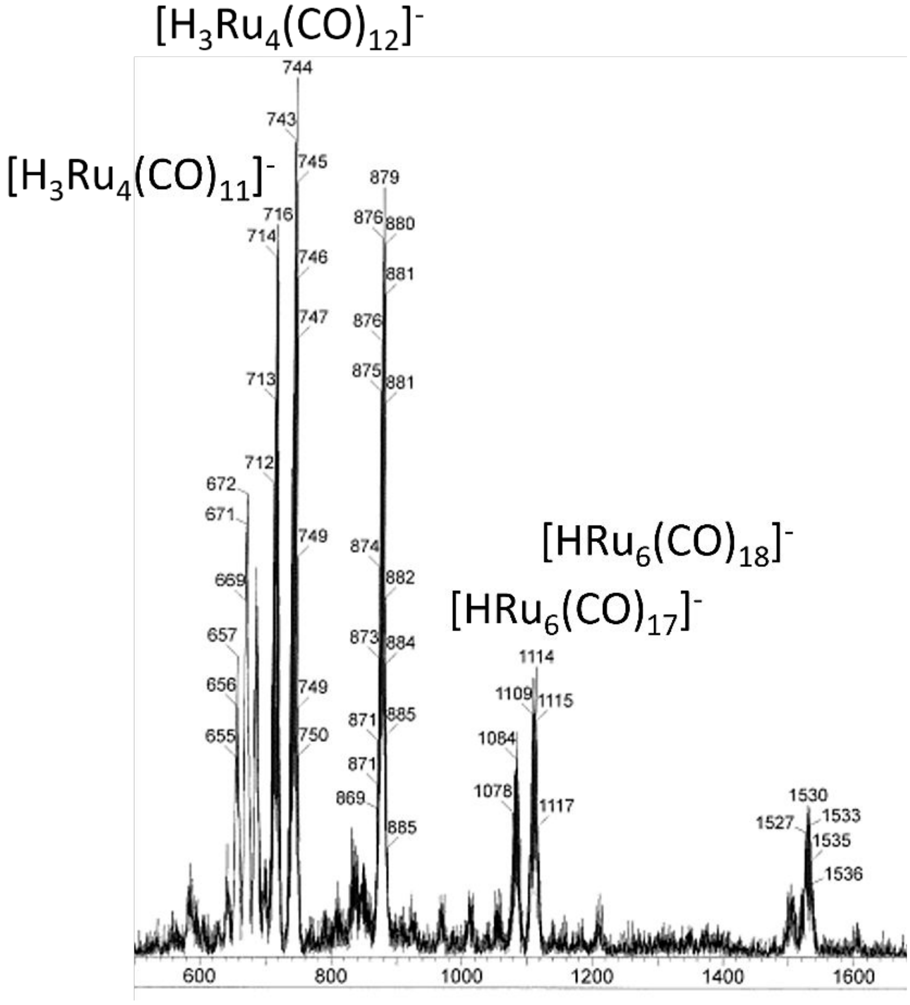

Figure S15. ESI-MS spectrum in CH_3OH (ES–) of the mixture at the end of the reactivity experiment: $[NEt_4][CuRu_6(CO)_{22}]$ (**2**) in ^{i}PrOH, for 24 h at 82 °C.

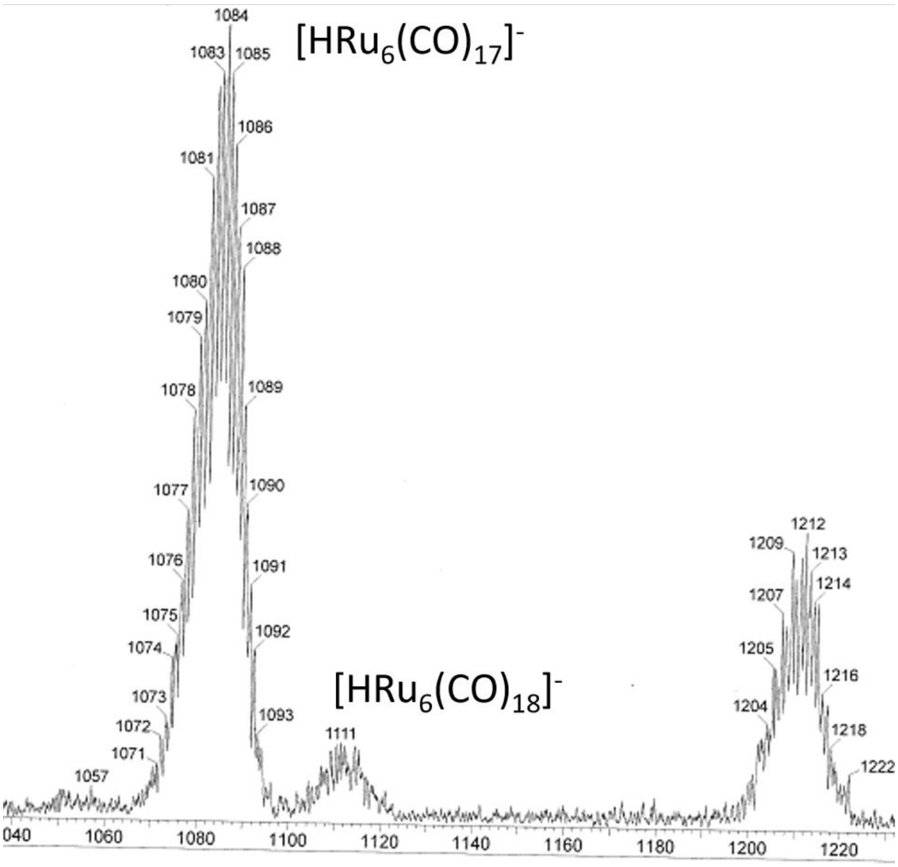

Figure S16. ESI-MS spectrum in CH_3OH (ES–) of the mixture at the end of the reactivity experiment: $[NEt_4][CuRu_6(CO)_{22}]$ (**2**) in ^{i}PrOH for 24 h at 82 °C, in the presence of substrate 4-Fluoroacetophenone, acetone extraction.

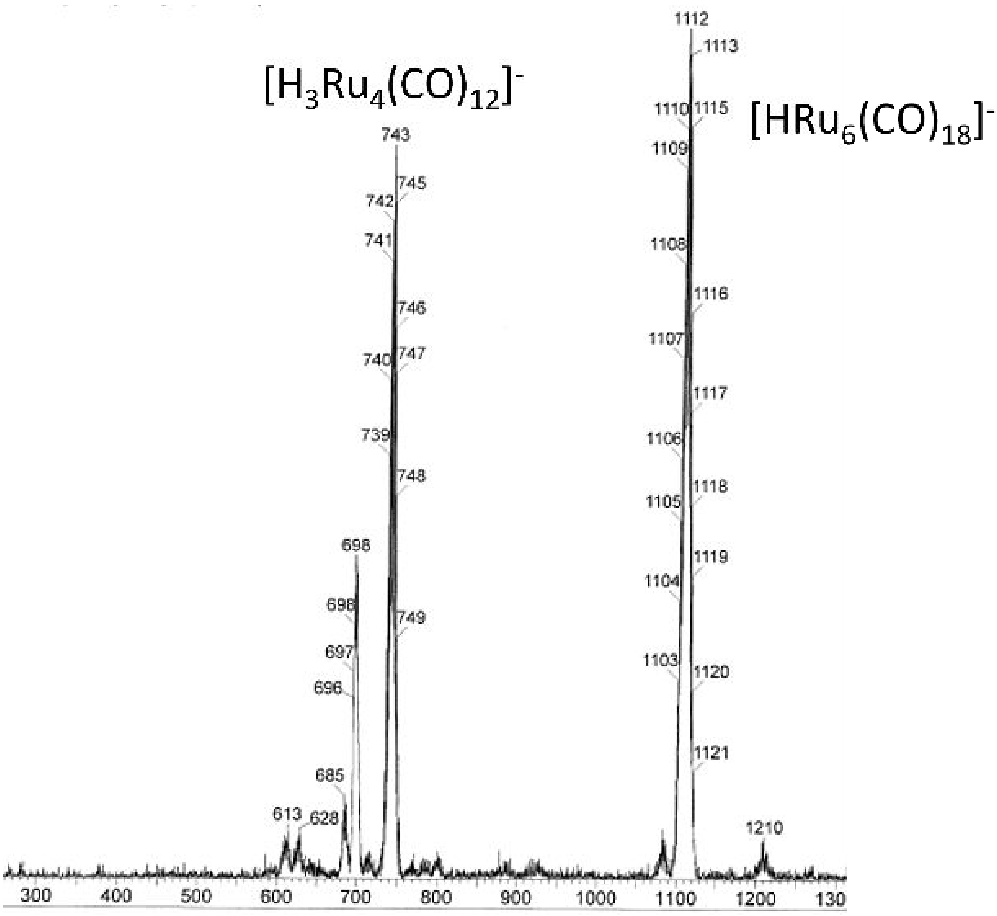

Figure S17. ESI-MS spectrum in CH_3OH (ES–) of the mixture at the end of the reactivity experiment: $[NEt_4][CuRu_6(CO)_{22}]$ (**2**) in ^{i}PrOH for 24 h at 82 °C, in the presence of substrate 4-Fluoroacetophenone, CH_2Cl_2 extraction.

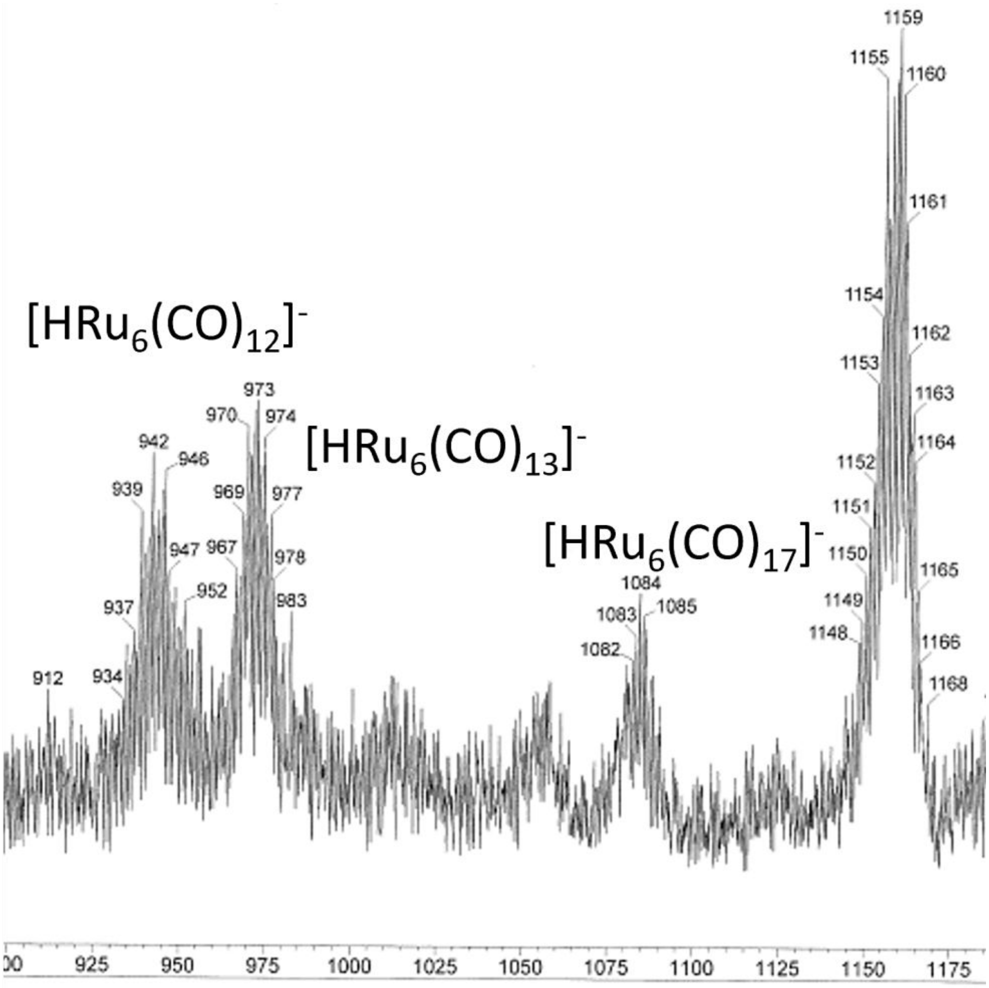

Figure S18. ESI-MS spectrum in CH_3OH (ES–) of the mixture at the end of the reactivity experiment: $[NEt_4][AgRu_6(CO)_{22}]$ (**2**) in ^{i}PrOH for 24 h at 82 °C.

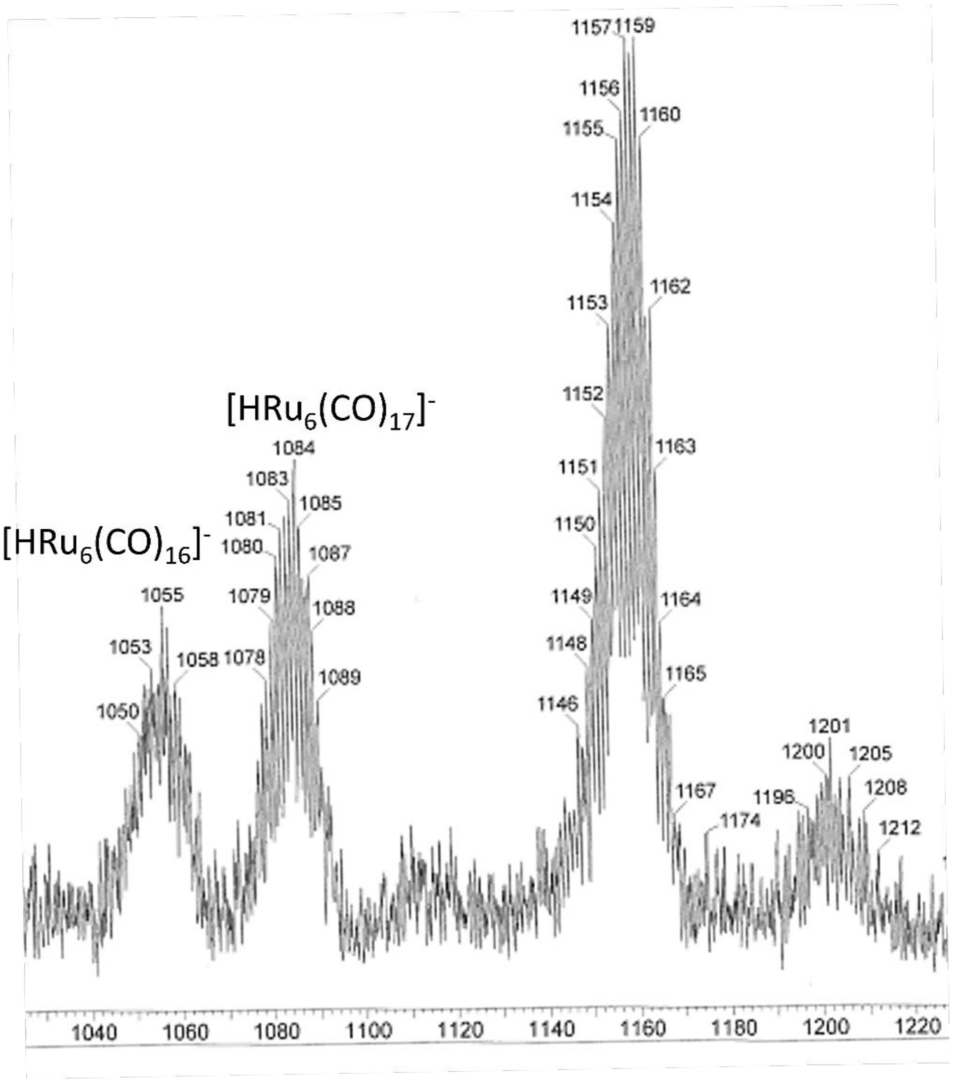

Figure S19. ESI-MS spectrum in CH_3OH (ES–) of the mixture at the end of the reactivity experiment: $[NEt_4][AgRu_6(CO)_{22}]$ (**3**) in ^{i}PrOH, for 24 h at 82 °C, in the presence of substrate 4-Fluoroacetophenone.

Table S1. Selected average computed data (a.u.) at M-M and M-H b.c.p. for $[Cu(\mu\text{-}H)_2\{Ru_3(CO)_{11}\}_2]^-$ (ρ = electron density; V = potential energy density; E = energy density; $\nabla^2\rho$= Laplacian of electron density) and Wiberg bond orders.

Bond	ρ	V	E	$\nabla^2\rho$	Wiberg b.o.
Cu-H	0.066	-0.074	-0.013	0.192	0.211
Ru-H	0.113	-0.124	-0.043	0.153	0.556
Cu-Ru	0.036	-0.031	-0.011	0.040	0.270
Ru(H)-Ru(Cu)	0.042	-0.031	-0.010	0.042	0.480
Ru(Cu)-Ru	0.046	-0.034	-0.013	0.029	0.524
Ru(H)-Ru	0.050	-0.038	-0.015	0.034	0.588

Table S2. Selected computed data (a.u.) at M-M b.c.p for **5** (ρ = electron density; V = potential energy density; E = energy density; $\nabla^2\rho$= Laplacian of electron density) and Wiberg bond orders. Please refer to Figure 10 for the numbering.

Bond	ρ	V	E	$\nabla^2\rho$	Wiberg b.o.
Cu(9)-Cu(10)	0.040	-0.044	-0.014	0.067	0.194
Cu(9)-Ru(1)	0.041	-0.038	-0.012	0.058	0.270
Cu(10)-Ru(5)	0.038	-0.035	-0.011	0.053	0.254
Cu(9)-Ru(2)	0.044	-0.040	-0.013	0.052	0.299
Cu(10)-Ru(7)	0.043	-0.039	-0.013	0.050	0.294
Cu(9)-Ru(3)	0.038	-0.031	-0.011	0.031	0.245
Cu(10)-Ru(6)	0.037	-0.029	-0.011	0.030	0.245
Cu(9)-Ru(5)	0.045	-0.042	-0.012	0.069	0.337
Cu(10)-Ru(1)	0.042	-0.039	-0.012	0.062	0.321
Ru(1)-Ru(2)	0.039	-0.027	-0.009	0.034	0.411
Ru(5)-Ru(7)	0.040	-0.029	-0.009	0.040	0.420
Ru(1)-Ru(3)	0.051	-0.047	-0.014	0.080	0.516
Ru(5)-Ru(6)	0.048	-0.042	-0.013	0.065	0.498
Ru(2)-Ru(3)	0.047	-0.041	-0.011	0.076	0.437
Ru(6)-Ru(7)	0.047	-0.042	-0.011	0.076	0.440
Ru(2)-Ru(4)	0.053	-0.040	-0.016	0.033	0.575
Ru(7)-Ru(8)	0.052	-0.039	-0.016	0.032	0.566
Ru(3)-Ru(4)	0.046	-0.035	-0.013	0.033	0.490
Ru(6)-Ru(8)	0.047	-0.035	-0.014	0.033	0.511
Ru(1)-Ru(4)	------	------	------	------	0.400
Ru(5)-Ru(8)	------	------	------	------	0.398

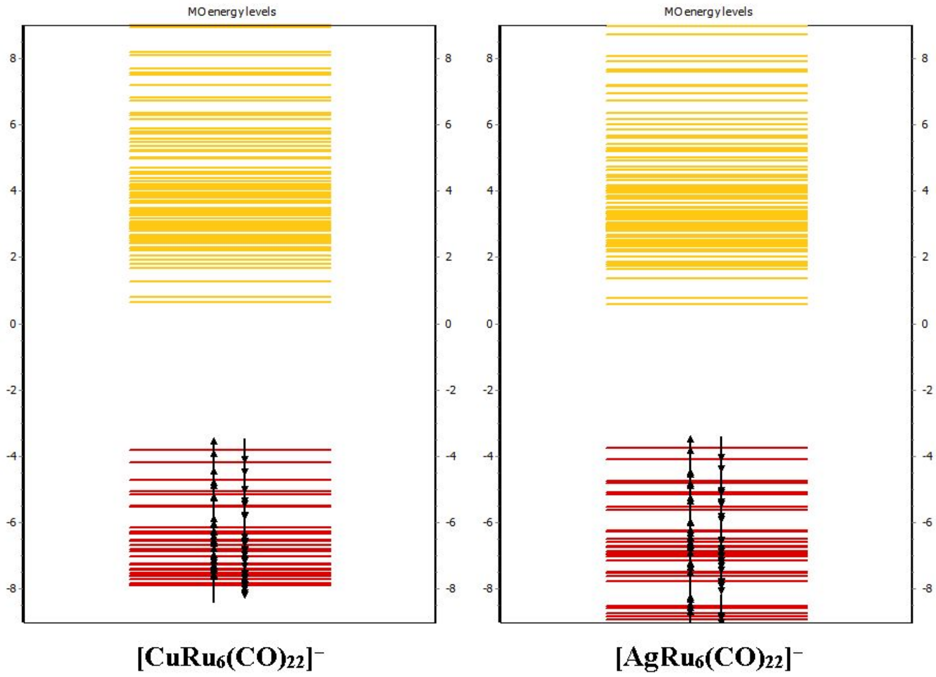

Figure S20. Molecular orbitals diagrams (PBEh-3c calculations) of clusters **2** and **3**. Energy values in eV.

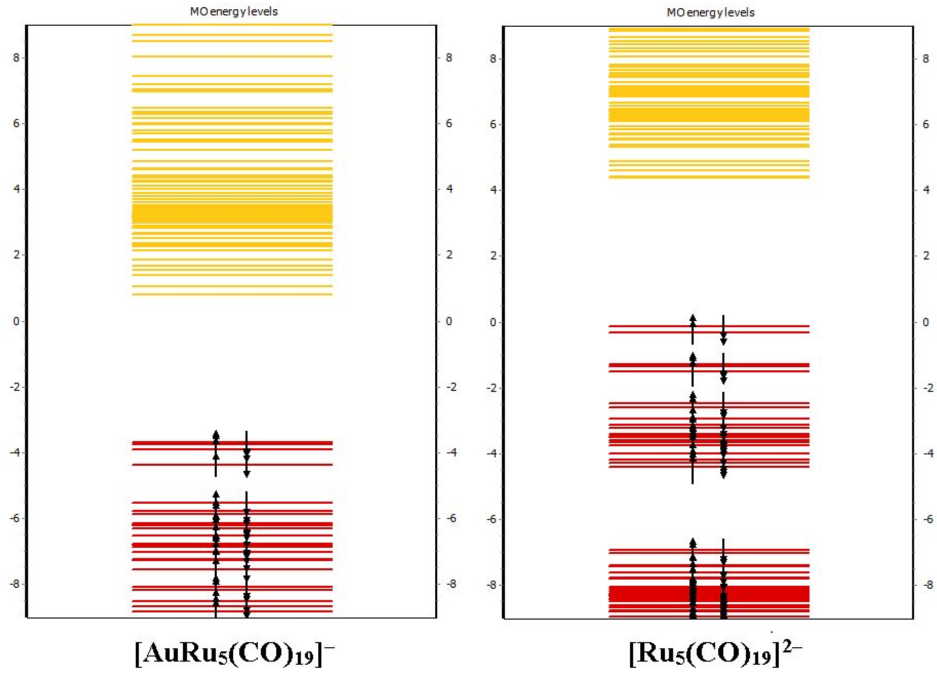

Figure S21. Molecular orbitals diagrams (PBEh-3c calculations) of clusters **4** and $[Ru_5(CO)_{19}]^{2-}$. Energy values in eV.

Table S3. Catalytic transfer hydrogenation of 4-fluoroacetophenone with heterometallic - Control Experiments.

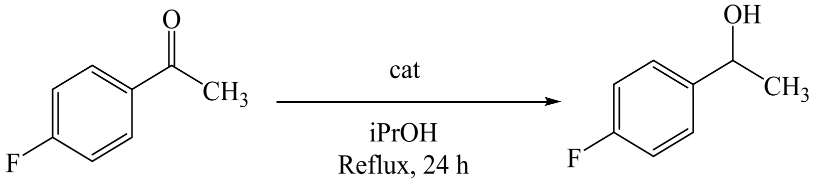

Entry	cat	cat (% mol/mol)	KO^{t}Bu (% mol/mol)	Conversion (%) 5 h	Conversion (%) 24 h
1-blanck	none	/	/	n.d.	n.d.
2-blanck		/	10	n.d.	n.d.
1-Ru	$RuCl_3\cdot 3H_2O$	1	/	n.d.	n.d.
1-Cu	$[Cu(CH_3CN)_4][BF_4]$	1	/	n.d.	n.d.
1-Ag	$AgNO_3$	1	/	n.d.	n.d.
1-Au	$Au(Et_2S)Cl$	1	/	n.d.	n.d.

General conditions: catalyst (3 µmol, 1% mol/mol), iPrOH (5 mL), KO^{t}Bu (10 mol% when added), 4-Fluoroacetophenone (36.5 µL, 300 µmol), T = 82°C, N_2 atmosphere; the conversions were determined by ^{19}F NMR spectroscopy.

Table S4. Crystal data and experimental details for $[NEt_4]$[**2**], $[NEt_4]$[**3**], $[NEt_4]$[**4**], $[NEt_4]_2$[**5**]·1.5CH_2Cl_2, **6**·solv, **9**·1.5toluene, $[NEt_4]$[**10**], $[NEt_4]_2$[**13**]·CH_2Cl_2, $[NEt_4]_2$[**13**]·CH_3COCH_3,

	**$[NEt_4]$[2]**	**$[NEt_4]$[3]**	**$[NEt_4]$[4]**	**$[NEt_4]$[4] (bis)**
Formula	$C_{30}H_{20}CuNO_{22}Ru_6$	$C_{30}H_{20}AgNO_{22}Ru_6$	$C_{27}H_{20}AuNO_{19}Ru_5$	$C_{27}H_{20}AuNO_{19}Ru_5$
Fw	1416.43	1460.76	1364.76	1364.76
T, K	100(2)	100(2)	100(2)	100(2)
λ, Å	0.71073	0.71073	0.71073	0.71073
Crystal system	Orthorhombic	Monoclinic	Monoclinic	Monoclinic
Space Group	*Aba*2	*C*2/*c*	*C*2/*c*	*C*2/*c*
a, Å	22.000(5)	13.3494(9)	20.473(3)	20.5278(16)
b, Å	42.583(11)	24.8442(17)	13.971(2)	13.9898(10)
c, Å	17.593(4)	12.9777(9)	14.254(2)	14.2842(11)
α, °	90	90	90	90
β, °	90	111.479(2)	115.568(3)	115.622(3)
γ, °	90	90	90	90
Cell Volume, $Å^3$	16481(7)	4005.2(5)	3678.0(11)	3698.8(5)
Z	16	4	4	4
D_c, g cm^{-3}	2.283	2.422	2.465	2.451
μ, mm^{-1}	2.729	2.766	6.053	6.019
F(000)	10816	2776	2560	2560
Crystal size, mm	0.18×0.16×0.15	0.16×0.13×0.10	0.14×0.12×0.10	0.18×0.13×0.11
θ limits, °	1.557-26.999	1.639-26.000	1.828–25.094	1.825–25.200
Index ranges	$-28 \le h \le 28$<br>$-54 \le k \le 54$<br>$-22 \le l \le 22$	$-16 \le h \le 16$<br>$-30 \le k \le 30$<br>$-16 \le l \le 16$	$-24 \le h \le 24$<br>$-16 \le k \le 16$<br>$-17 \le l \le 17$	$-24 \le h \le 24$<br>$-16 \le k \le 16$<br>$-17 \le l \le 17$
Reflections collected	86091	28033	17223	17623
Independent reflections	17904 [R_{int} = 0.0732]	3945 [R_{int} = 0.0322]	3274 [R_{int} = 0.0525]	3325 [R_{int} = 0.0525]
Completeness to θ max	99.9%	99.9%	99.8%	99.9%
Data / restraints / parameters	17904 / 512 / 1155	3945 / 62 / 309	3274 / 276 / 247	3325 / 341 / 294
Goodness on fit on F^2	1.204	1.270	1.383	1.338

R_1 (I > 2σ(I))	0.0552	0.0187	0.1209	0.1161
wR_2 (all data)	0.1029	0.0433	0.2763	0.2683
Largest diff. peak and hole, e $Å^{-3}$	1.877 / -1.093	0.393 / -0.624	2.726 / –4.292	2.726 / –3.534

	**$[NEt_4]_2[5]·1.5CH_2Cl_2$**	**6·solv**	**9·1.5toluene**
Formula	$C_{43.5}H_{43}Cl_3Cu_2N_2O_{26}Ru_8$	$C_{84}H_{60}Cu_4O_{12}P_4Ru_4$	$C_{71.5}H_{59}O_8P_3Ru_3$
Fw	2051.79	2043.64	1442.30
T, K	100(2)	100(2)	100(2)
λ, Å	0.71073	0.71073	0.71073
Crystal system	Monoclinic	Orthorhombic	Triclinic
Space Group	$P2_1/c$	*Cmcm*	$P\bar{1}$
a, Å	22.6835(15)	22.783(3)	11.2343(6)
b, Å	22.2726(13)	16.220(2)	12.8686(6)
c, Å	12.8510(8)	26.734(3)	22.6451(11)
α, °	90	90	89.278(2)
β, °	103.453(2)	90	80.053(2)
γ, °	90	90	85.804(2)
Cell Volume, $Å^3$	6314.4(7)	9879(2)	3215.9(3)
Z	4	4	2
D_c, g cm^{-3}	2.158	1.374	1.489
μ, mm^{-1}	2.719	1.551	0.825
F(000)	3948	4048	1458
Crystal size, mm	0.15×0.12×0.09	0.25×0.19×0.09	0.21×0.18×0.14
θ limits, °	1.829–25.400	1.541–25.999	1.587–26.444
Index ranges	$-27 \le h \le 27$<br>$-26 \le k \le 26$<br>$-15 \le l \le 15$	$-28 \le h \le 28$<br>$-19 \le k \le 20$<br>$-32 \le l \le 32$	$-14 \le h \le 14$<br>$-16 \le k \le 16$<br>$-28 \le l \le 28$
Reflections collected	74672	48482	51263
Independent reflections	11569 [R_{int} = 0.0827]	5085 [R_{int} = 0.0855]	13180 [R_{int} = 0.0746]
Completeness to θ max	99.6%	99.9%	99.9%
Data / restraints / parameters	11569 / 435 / 793	5085 / 480 / 375	13180 / 42 / 730
Goodness on fit on F^2	1.253	1.178	1.080

R_1 (I > 2σ(I))	0.1000	0.0499	0.0526
wR_2 (all data)	0.2211	0.1014	0.1252
Largest diff. peak and hole, e $Å^{-3}$	6.008 / –4.205	0.996 / –1.2865	1.668 / –0.977

	**[NEt$_4$][10]**	**[NEt$_4$]$_2$[13]·CH$_2$Cl$_2$**	**[NEt$_4$]$_2$[13]·CH$_3$COCH$_3$**
Formula	$C_{19}H_{21}NO_{12}Ru_3$	$C_{35}H_{42}Cl_2N_2O_{18}Ru_6$	$C_{37}H_{46}N_2O_{19}Ru_6$
Fw	758.58	1456.02	1429.18
T, K	100(2)	100(2)	100(2)
λ, Å	0.71073	0.71073	0.71073
Crystal system	Monoclinic	Monoclinic	Triclinic
Space Group	$P2_1/c$	$P2_1/n$	$P\bar{1}$
a, Å	13.2567(3)	9.6573(15)	10.9562(12)
b, Å	11.7246(3)	19.893(3)	12.0771(13)
c, Å	17.0204(4)	23.957(4)	18.628(2)
α, °	90	90	95.820(4)
β, °	105.6020(10)	91.267(4)	90.883(4)
γ, °	90	90	108.142(4)
Cell Volume, $Å^3$	2547.99(11)	4601.3(12)	2327.4(4)
Z	4	4	2
D_c, g cm^{-3}	1.977	2.102	2.039
μ, mm^{-1}	1.815	2.108	1.972
F(000)	1480	2832	1396
Crystal size, mm	0.15×0.12×0.09	0.19×0.12×0.10	0.21×0.16×0.11
θ limits, °	1.595–26.996	1.700–25.098	1.786–25.0478
Index ranges	-16 ≤ h ≤ 16<br>-14 ≤ k ≤ 14<br>-21 ≤ l ≤ 21	-11 ≤ h ≤ 11<br>-23 ≤ k ≤ 23<br>-28 ≤ l ≤ 28	-13 ≤ h ≤ 13<br>-14 ≤ k ≤ 14<br>-22 ≤ l ≤ 22
Reflections collected	52385	52825	12773
Independent reflections	5544 [R_{int} = 0.1056]	8012 [R_{int} = 0.0895]	8035 [R_{int} = 0.0597]
Completeness to θ max	100.0%	97.6%	97.5%
Data / restraints / parameters	5544 / 2506 / 3897	8012 / 534 / 568	8035 / 198 / 587
Goodness on fit on F^2	1.241	1.222	1.127
R_1 (I > 2σ(I))	0.0393	0.0864	0.0971

wR_2 (all data)	0.06833	0.1898	0.2484
Largest diff. peak and hole, e Å^{-3}	0.616 / –1.001	4.449 / –1.861	2.975 / –2.847
